# Sleep, Steps, and Screens: Between- and within-person effects of digital markers of daily life behaviors on smartphone-based assessments of cognitive functioning in depression

**DOI:** 10.1016/j.nsa.2026.106985

**Published:** 2026-02-14

**Authors:** Marcos Ross-Adelman, George Aalbers, Faith Matcham, Daniel Leightley, Carolin Oetzmann, Ewan Carr, Sara Siddi, Josep M. Haro, Peter Annas, Maria Dalby, Vaibhav A. Narayan, Matthew Hotopf, Inez Myin-Germeys, Femke Lamers, Brenda W.J.H. Penninx, RADAR-CNS consortium

**Affiliations:** a Department of Psychiatry, Amsterdam UMC, Vrije Universiteit Amsterdam, the Netherlands; b Amsterdam Public Health, Mental Health Program, Amsterdam, the Netherlands; c Department of Psychological Medicine, Institute of Psychiatry, Psychology and Neuroscience, King's College London, United Kingdom; d School of Psychology, University of Sussex, Falmer, United Kingdom; e School of Life Course and Population Sciences, Faculty of Life Sciences and Medicine, King's College London, United Kingdom; f Department of Biostatistics & Health Informatics, Institute of Psychiatry, Psychology and Neuroscience, King's College London, United Kingdom; g Parc Sanitari Sant Joan de Déu, Sant Joan de Déu Research Institute (IRSJD), CIBERSAM, Barcelona, Spain; h H. Lundbeck A/S, Valby, Denmark; i Muna Therapeutics, Copenhagen, Denmark; j Davos Alzheimer's Collaborative (DAC), Geneva, Switzerland; k South London and Maudsley NHS Foundation Trust, London, United Kingdom; l Department of Neurosciences, Center of Contextual Psychiatry, KU Leuven, Belgium

**Keywords:** Major depressive disorder, Cognitive functioning, Digital health, Ambulatory assessment, Patient monitoring

## Abstract

Cognitive impairment represents a core feature of major depressive disorder (MDD), often persisting after mood symptoms remit and not addressed by usual antidepressant treatments. Despite its relevance, cognition is typically assessed with infrequent tests in clinical settings, overlooking its contextual nature. Smartphones and wearables enable ecologically valid, repeated measurements of cognition and daily life behaviors that may impact it. We examined whether sleep duration, step count, and smartphone screen time are associated with cognitive functioning in MDD.

We conducted secondary analyses of RADAR-MDD, a multicenter study following individuals with recurrent MDD. Cognitive functioning – self-reported and performance-based – was assessed with the THINC-it® app. Sleep duration and step count were measured with Fitbit devices, and screen time with the RADAR-Base app. Cognitive assessments (outcomes) were linked to behavioral measures (predictors) from the day of and the day preceding each assessment. Two-level multilevel models estimated between-person (differences in participant means) and within-person (deviations from participant means) effects. The sample included 502 participants, further subdivided by behavior–cognitive outcome pair.

For performance-based cognitive assessments, positive associations at the between-person level were found for step count (β = 0.104, SE = 0.031, p < 0.001) and screen time (β = 0.075, SE = 0.036, p = 0.038), and sleep duration showed a quadratic negative effect (β = −0.080, SE = 0.018, p < 0.001). No within-person effects were detected. For self-reported cognitive functioning, step count showed positive associations both between (β = 0.161, SE = 0.037, p < 0.001) and within persons (β = 0.027, SE = 0.010, p = 0.005), while screen time was negatively associated within persons (β = −0.033, SE = 0.011, p = 0.002).

Our findings illustrate that smartphones and wearables can collect meaningful daily life data of MDD patients that can be used to support cognitive health. Step count emerges as a promising behavioral target as it is simple to track and is correlated with better cognitive outcomes.

## 1 Introduction

Smartphones and wearables are increasingly used in psychiatry to capture information about people's daily lives that can improve monitoring of patients with depression (De Angel et al., 2022; Fedor et al., 2023; Leaning et al., 2024; Trull and Ebner-Priemer, 2020). Through built-in sensors and app-based logging, mobile technologies can passively capture behavioral, physiological, and contextual data (i.e., digital phenotyping; Onnela and Rauch, 2016), while short surveys and tasks can provide valuable insight into the patient's experience of disease and cognitive functioning (i.e., ecological momentary assessments; Shiffman et al., 2008). In short, these everyday devices enable high-frequency, ecologically valid, multimodal data collection (Torous et al., 2021). The current study explores how digital markers of daily life behaviors – sleep duration, step count, and smartphone screen time – relate to smartphone-based assessments of cognitive functioning in individuals with recurrent major depressive disorder (MDD).

Cognitive impairment is a central feature of MDD (Ahern and Semkovska, 2017; American Psychological Association, 2013) that often persists even after mood symptoms improve and not is addressed by usual antidepressant treatments, contributing to poor functioning and increased relapse risk (Conradi et al., 2011; Matcham et al., 2022b; Semkovska et al., 2019). Despite its clinical relevance, cognition is typically assessed using long, infrequent tests in controlled settings. Cognition, however, is dynamic and contextual, shaped by multiple factors that unfold in daily life. Mobile technologies can identify how these factors are linked to cognition (Weizenbaum et al., 2020), and may be used to inform interventions aimed at supporting cognitive health in MDD.

We focus on three digital markers of daily life behaviors linked to both depression and cognitive functioning: sleep duration, step count, and smartphone screen time. Sleep difficulties are central to MDD diagnosis (American Psychological Association, 2013) and prior work has shown that lower wearable-derived sleep duration is linked to worse depression outcomes (Matcham et al., 2024). Moreover, better information processing measured with a smartphone-based cognitive task has been linked to higher wearable-derived sleep duration (Kalanadhabhatta et al., 2021). Higher step count, a proxy for physical activity, has been related to reduced risk of depression (Bizzozero-Peroni et al., 2024) and improved cognitive functioning (Calamia et al., 2018; Rojer et al., 2021). Smartphone use, in contrast, is less well understood. While overuse is associated with distraction and task-delay (Aalbers et al., 2022; Siebers et al., 2024), some types of engagement (e.g., information seeking, gaming) may support cognitive functioning (Wilmer et al., 2017). Problematic smartphone use has been linked to depression, though there is still debate regarding the direction of causality (Cheng and Meng, 2021; Elhai et al., 2017). By examining the relationship between these daily life behaviors and cognitive functioning, we aim to advance the understanding of cognition in depression using data gathered with commonplace mobile technologies.

We also seek to leverage the capacity that mobile technologies have for repeated and remote measurement. Prior research has largely focused on cross-sectional and/or between-person analyses. However, smartphones and wearables can collect vast amounts of data, enabling the detection of within-person changes that could improve personalized monitoring (Ross-Adelman et al., 2025; Trull and Ebner-Priemer, 2014). To fully realize the utility of these devices, the data they generate must extend beyond characterizing average differences between people; they must also show within-person associations with an outcome of interest (Hamaker, 2012; Hamaker and Wichers, 2017; Vaughan and Birney, 2023). This means determining whether changes in sleep, step count, and screen time co-vary with changes in cognitive functioning within individuals over time.

This study uses longitudinal data from the RADAR-MDD study, a multicenter cohort that followed over 600 individuals with recurrent depression for an average of 18 months (Matcham et al., 2019; Matcham et al., 2022a). Sleep duration, step count, and smartphone use were passively captured via built-in wearable and phone apps. Cognitive functioning was assessed with THINC-it®, a smartphone app developed for MDD that combines self-report (i.e., how people experience their own functioning) and performance-based (i.e., cognitive tasks) measures (McIntyre et al., 2017). We hypothesize that sleep duration and step count will show positive associations with both cognitive measures at both the between- and within-person level. Given mixed evidence, we expect associations for screen time but make no directional prediction.

## 2 Methods

### 2.1 Sample

This paper presents a secondary analysis of RADAR-MDD, a multicenter longitudinal observational cohort study designed to remotely monitor individuals with recurrent major depressive disorder using smartphones and wearables (Matcham et al., 2019). The cohort included 623 individuals (75% female, 79% white, mean age = 46.4 years, range: 23-84) recruited in the Netherlands, Spain, and the United Kingdom. Participants had a lifetime history of recurrent MDD, provided regular self-report and performance-based assessments through a dedicated smartphone app, and wore a Fitbit Charge 2 or 3 for passive data collection. They were followed for an average of 18 months (range: 11-24), with a median of 541 days (Matcham et al., 2022a). Voluntarily providing data after the study officially was also possible, and was approved by the corresponding ethics committee. Inclusion criteria were: meeting DSM-5 criteria of MDD, at least two lifetime depressive episodes (one within two years prior to enrollment), aged over 18 years, fluent in English, Dutch, Catalan, or Spanish, willing to use an Android smartphone and Fitbit during the study, and able to provide informed consent. Exclusion criteria included a history of bipolar disorder, schizophrenia, schizoaffective disorder, MDD with psychotic features, dementia, or a medical condition likely to impede daily functioning for more than two consecutive weeks (Matcham et al., 2019). Full details on RADAR-MDD recruitment, data availability, and retention are reported elsewhere (Matcham et al., 2022a). For this analysis, we used a subset of 502 participants who provided assessments on cognitive functioning that could be linked to sleep duration, step count, and smartphone screen time data.

### 2.2 Measures

#### 2.2.1 Cognitive functioning (outcomes)

Cognition was assessed using the THINC-it® app, a self-administered mobile tool validated for use in MDD that takes 10-15 min to complete (McIntyre et al., 2017; THINC-it physician guide, 2017). Participants were trained once at baseline and then received push notifications every six weeks to complete the tasks. THINC-it® includes five modules. One is the 5-item Perceived Deficit Questionnaire (PDQ-5; Sullivan et al., 1990) which assesses the self-reported experience of cognitive functioning in a 5-point Likert scale. Items were summed into a score ranging from 0 to 20 and reversed so that lower values indicate poorer perceived cognitive functioning. The remaining four are performance-based tasks: *Spotter* (‘Choice Reaction Time’ task; measures attention via mean reaction time of correct responses), *Symbol Check* (‘1-back’ task; measures working memory via number of correct responses), *Codebreaker* (‘Digit Symbol Substitution’ test; measures processing speed via number of correct responses), and *Trails* (the ‘Trails Making Test B’; measures attention switching via completion time). Because task scores were moderate to highly correlated (Supplementary Fig. 1, Appendix, section A), and to reduce multiple testing, we created a normalized 0-100 global performance score to reflect a summary of the four tasks, with higher scores indicating better performance (see section 2.3. Data preprocessing). PDQ-5 and the global performance scores served as separate outcomes in the analysis because they contribute independently to patient functioning (McIntyre et al., 2017) and they are not highly correlated (Supplementary Fig. 1, Appendix, section A). All THINC-it® modules have been validated against paper and pencil versions (McIntyre et al., 2017), have shown moderate to high levels of test-retest reliability (Dalby et al., 2022; Harrison et al., 2018) and are sensitive to change in cognitive functioning in adults with MDD (McIntyre et al., 2020). Internal consistency coefficients (i.e., McDonald's hierarchical omega, ω_h_; Kalkbrenner, 2023) for the global performance score and the PDQ-5 score indicate sufficient internal consistency (ω_h_ ≈ 0.8 for both scores; find more on McDonald's omega in Appendix, section C).

#### 2.2.2 Behavioral measures (predictors)

We used: *(a) Sleep duration.* Total daily sleep hours (sum of “non-awake” stages), measured by the Fitbit Charge 2/3. *(b) Step count.* Total daily steps within the day, measured by the Fitbit Charge 2/3. *(c) Scree time (smartphone use)*. Total daily minutes with phone unlocked, recorded continuously with the RADAR-Base app (Ranjan et al., 2019). Behavioral data from the day of- and the day before each cognitive assessment was used (see section 2.3. Data preprocessing). Fitbit Charge 2 and 3 show good validity to assess sleep duration (de Zambotti et al., 2018; Eylon et al., 2023; Haghayegh et al., 2019), but tend to overestimate step count (Bai et al., 2021; Hargis et al., 2018; Irwin and Gary, 2022).

#### 2.2.3 Covariates (demographic variables)

Age, gender, and years of education were included as covariates given their influence on cognitive functioning (Angrisani et al., 2020; Lövdén et al., 2020; Murman, 2015).

### 2.3 Data preprocessing

Data preprocessing was conducted using *R* (version 4.4.3). We first removed duplicate observations, excluded participants who withdrew early, and discarded cognitive assessments that were less than one week apart (participants sometimes self-initiated tasks outside of the intended design such as completing them repeatedly within a day or week; see Appendix, section D for more details on adherence). Each variable was preprocessed separately before merging them. Outliers were addressed using domain knowledge and winsorization, consistently applied at ±3 standard deviations from the mean (see Appendix, section E for details on handling outliers).

For consistency, cognitive measures scores were coded so that higher values reflect better performance/perception (i.e., *Spotter* and *Trails* task scores were reversed). Subsequently, a normalized global cognitive performance score was created whenever a participant completed two or more tasks at any given measurement occasion (≈95% of occasions). Individual task scores were first normalized to a 0–100 scale using the observed minimum and maximum values for that task across the sample ((*X* – min)/(max – min) x 100). Per measurement occasion, a global score was then calculated as the mean of their normalized task scores. For occasions when participants completed only one task (≈5% of occasions), their normalized score for that task was used as their global score.

Cognitive assessments were linked to behavioral data from the same day and the preceding day. If both days were available, a mean was taken; if only one, that day was used. This two-day window was selected based on the assumption that proximal associations are stronger than distal ones. In addition, we found that these two-day measures of sleep duration, step count, and screen time were highly correlated with measures taken from a week preceding the cognitive assessment (correlations between 0.775 and 0.925). The two-day window also had the advantage of larger sample sizes due to less missingness.

### 2.4 Data analysis

Analyses were performed in R (version 4.4.3) using the *lme4* (version 1.1-37) and *lmerTest* (version 3.1-3) packages. We estimated two-level multilevel models (i.e., participants and repeated measures nested within participants) using maximum likelihood estimation (MLE). For each behavioral predictor (sleep duration, step count, screen time), two models were estimated – one predicting PDQ-5 and one predicting global performance – leading to six models in total. Models were adjusted for age, gender, and years of education. To account for potential non-linear effects (i.e., the possibility that both too little and too much sleep may negatively impact cognitive functioning), two additional models including a quadratic term for sleep duration were estimated for both cognitive outcomes.

Due to the nested structure of the data, predictors were person-mean centered to separate within- and between-person effects (Wang and Maxwell, 2015). Each behavioral measure was separated into a level-2 component (i.e., individual means that capture between-person differences) and a level-1 component (i.e., deviations from the mean over time that capture differences within a person). Both components were separately entered in the model, allowing for simultaneous and independent estimation of both the within-person and between-person effects of the predictor on the outcome (Wang and Maxwell, 2015).

For each behavioral predictor – cognitive outcome pair, model estimation started with an intercept-only model to compute intraclass correlations (ICC) and identify the proportion of variance in each level. To account for the non-independence of repeated measures nested within participants, random intercepts were specified (Gelman and Hill, 2006; see Supplementary Figs. 6 and 7, Appendix, section G, for baseline variation across participants). Model complexity was gradually increased by first testing the effect of time (relative to each participant's start date), then adding the within- and between-person components of the behavioral predictor, and finally the demographic variables. Because models were nested within each other, model fit was statistically assessed using deviance comparisons. Adding random slopes for the behavioral predictors did not improve fit.

Missing data were handled using listwise deletion of missing observations, the standard approach for multilevel modelling (Nezlek and Mroziński, 2020). Predictors and outcomes were standardized (mean = 0, SD = 1), and standardized β coefficients are reported for comparability across models. Multiple comparisons were adjusted with a Bonferroni-corrected alpha of 0.0083 (0.05/6). Unstandardized coefficients are reported in the OSF project URL under ‘Results → Main Analysis’ (https://osf.io/r36mk/). Multilevel modelling assumptions were checked and met (find in main analysis code).

#### 2.4.1 Sensitivity analyses

Given the associations found between cognitive functioning and depression severity found in prior work (Ross-Adelman et al., 2025), we tested whether the observed associations were independent from current depression levels. To this end, we ran a sensitivity analysis including depression severity scores that were temporally aligned with each behavioral predictor-cognitive outcome pair (see Appendix, section H).

Furthermore, although using a two-day window of behavioral data provided a larger sample size, we also tested whether associations would remain stable by running the models using one week of behavioral data preceding each cognitive assessment (see Appendix, section I).

Code for preprocessing, main and sensitivity data analysis is available in the OSF project URL (https://osf.io/r36mk/).

## 3 Results

### 3.1 Sample characteristics and descriptive statistics

The total sample consisted of 502 participants (75.3% female, mean age = 46, range: 18-80, mean years of education = 16, see Table 1). Mean follow-up time was approximately 9 months (SD = 8, range: 0-31). Preprocessing yielded 3050 PDQ-5 and 3052 global performance assessments (mean per participant = 6), which we linked to 158 632 sleep (mean per participant = 274 recorded days), 209 789 step count (mean per participant = 370 days), and 154 129 smartphone use days (mean per participant = 262 days). More details on preprocessing decisions and data availability can be found in the Appendix (sections D and E),

**Table 1:** Descriptive statistics of the used measures.

Baseline demographic factors (n = 502)	Mean	S.D.	Range
Female/Male gender (%)	378 females/124 males (75.3% female)		
Age	46	15	18 - 80
Years of education	16	5	0 - 36

Smartphone and wearable derived data	Mean	Between-person S.D.	Within-person S.D.	Median	Range
**Cognitive functioning**					
Averaged global performance score (t = 2700)					
Normalized scores	66	16.2	6.7	66.4	0 - 99.6
Averaged PDQ-5 (t = 2701)					
Sum score	10.3	4.9	2.2	10.4	0 - 20
**Behavioral measures**					
Averaged sleep duration (t = 2075)					
Sleep duration (hours)	7.5	1.2	1.1	7.5	2.8 - 12.8
Averaged step count (t = 2178)					
Step count (# steps)	6584	3591	2660	6483	112 - 30000
Averaged screen time (t = 1741)					
Unlock duration time (minutes)	175.8	120.2	78.4	172.9	10 - 633.7

On average, participants provided 5 cognitive assessments that could be linked to a behavioral measure (median = 4, range: 1-33), which were on average 80 days apart (SD = 86, median = 69 days). Assessments were most frequently completed in the afternoon (56.1%, 12:00-17:59), followed by the morning (24.2%, 7:00-11:59) and evening (17.4%, 18:00-23:59). A small proportion of assessments (2.3%) were completed during late-night and early-morning hours (00:00–06:59) (see Supplementary Fig. 2, Appendix, section B for a distribution on completion throughout the day). For modeling purposes, the sample was subdivided into six datasets depending on the behavioral predictor and cognitive functioning outcome combination, ranging from 418 to 441 participants and 1725 to 2166 observations (see Supplementary Fig. 5, Appendix, section F for a flowchart of sample selection).

Across all measures (except for sleep duration), between-person variability was larger than within-person variability. Time series plots showing the trajectories of the variables over time across participants can be found in Supplementary Figs. 6 and 7 (Appendix, section G). Performance-based cognitive functioning minimally improved over time (β = 0.011, SE = 0.001, p < 0.001, 95% CI: 0.009, 0.012), an expected finding reported in the THINC-it® user guide (*THINC-it physician guide*, 2017) and in previous work with RADAR-MDD data (Ross-Adelman et al., 2025).

### 3.2 The association between daily life behaviors and cognitive functioning in MDD

For step count and screen time, the best-fitting models included the within- and between-person components plus covariates. For sleep duration, the best-fitting model additionally included the quadratic terms. This was the case for both cognitive outcomes.

#### 3.2.1 Daily life behaviors and performance-based cognitive functioning

**Fig. 1:**
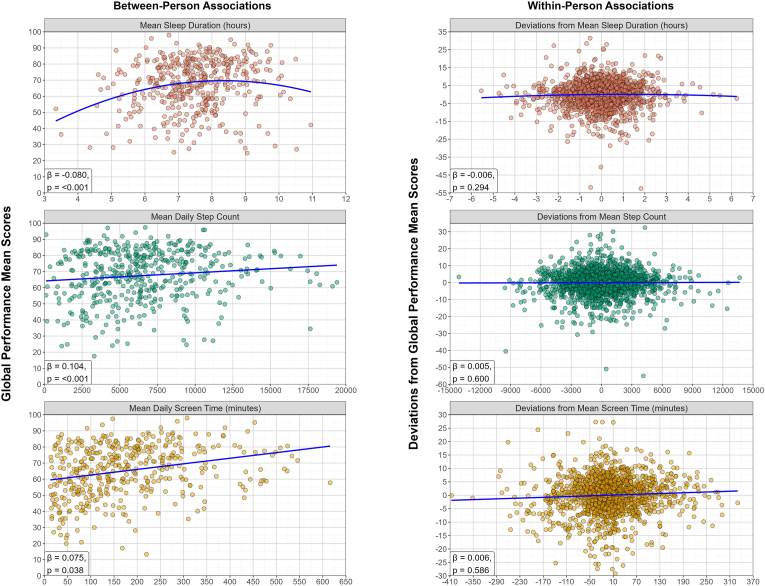
Panel plot illustrating the associations between digital markers of daily life behavior (sleep duration, step count and screen time) and smartphone-based assessments of performance-based cognitive functioning (i.e., global performance score of THINC-it® tasks). The left column contains the associations at the between-person level and includes participants' mean scores of the variables (n = 422 for sleep duration plot, n = 439 for step count plot, n = 418 for screen time plot). The right column contains the associations at the within-person level and includes the deviations from mean scores (t = 2063 for sleep duration plot, t = 2162 for step count plot, t = 1729 for screen time plot). Blue lines represent the average trend (quadratic for sleep duration, linear for step count and screen time). The standardized estimates (β) and significance (p) are shown at the bottom left corner of each plot. Results have been adjusted for age, gender and years of education.

Sleep duration, step count and screen time were separately modelled as predictors of performance-based cognitive functioning. The null models yielded ICCs of 0.786-0.791, indicating that around 79% of the variance in performance-based cognitive functioning is due to between-person differences, while the remaining 21% is due to within-person changes. Table 2 contains all standardized estimates and results are illustrated in Fig. 1. Unstandardized estimates to see relationships between variables in their original units are provided in the Appendix (section J).

**Table 2:** Standardized associations between digital markers of daily life behaviors (sleep duration, step count, and screen time) as predictors of performance-based cognitive smartphone assessments (global performance score of THINC-it® tasks). Level-1 is the within-person level. Level-2 is the between-person level. Significant associations are in bold. Abbreviations: N = unique participants, t = observations.

Model (predictor – outcome)	Sleep duration – Global Performance score (N = 422, t = 2063)			Step count – Global Performance score (N = 439, t = 2162)			Screen time – Global Performance score (N = 418, t = 1729)		
Fixed effects coefficients	Estimate (SE)	95% CI	p value	Estimate (SE)	95% CI	p value	Estimate (SE)	95% CI	p value
Mean/Intercept	**1.336 (0.177)**	**[0.990, 1.684]**	**<0.001∗∗∗**	**1.142 (0.167)**	**[0.814, 1.470]**	**<0.001∗∗∗**	**1.039 (0.182)**	**[0.683, 1.397]**	**<0.001∗∗∗**
Time (relative to each person's start)	**6.7E-04 (6.6E-05)**	**[5.4E-04, 8.0E-04]**	**<0.001∗∗∗**	**5.5E-04 (6.1E-05)**	**[4.3E-04, 6.7E-04]**	**<0.001∗∗∗**	**7.6E-04 (7.7E-05)**	**[6.1E-04, 9.1E-04]**	**<0.001∗∗∗**
Behavior within-person component	0.009 (0.011)	[-0.012, 0.029]	0.404	0.005 (0.010)	[-0.015, 0.025]	0.600	0.006 (0.012)	[-0.016, 0.029]	0.586
Behavior within-person component (quadratic)	−0.006 (0.005)	[-0.016, 0.005]	0.294	**-**	**-**	**-**	**-**	**-**	**-**
Behavior between-person component	0.026 (0.031)	[-0.036, 0.088]	0.412	**0.104 (0.031)**	**[0.042, 0.165]**	**<0.001∗∗∗**	**0.075 (0.036)**	**[0.004, 0.146]**	**0.038∗**
Behavior between-person component (quadratic)	**−0.080 (0.018)**	**[-0.116, -0.045]**	**<0.001∗∗∗**	**-**	**-**	**-**	**-**	**-**	**-**
Age	**−0.040 (0.002)**	**[-0.044, -0.036]**	**<0.001∗∗∗**	**−0.040 (0.002)**	**[-0.044, -0.036]**	**<0.001∗∗∗**	**−0.039 (0.002)**	**[-0.043, -0.034]**	**<0.001∗∗∗**
Gender (ref = male)	−0.080 (0.083)	[-0.243, 0.083]	0.338	−0.021 (0.078)	[-0.173, 0.132]	0.791	−0.040 (0.084)	[-0.205, 0.124]	0.629
Years of education	**0.031 (0.006)**	**[0.019, 0.043]**	**<0.001∗∗∗**	**0.037 (0.006)**	**[0.025, 0.049]**	**<0.001∗∗∗**	**0.039 (0.007)**	**[0.026, 0.052]**	**<0.001∗∗∗**

Random effects coefficients	Estimate (SD)	Estimate (SD)	Estimate (SD)
Level-2 error term	0.396 (0.629)	0.402 (0.634)	0.441 (0.664)
Level-1 error term	0.228 (0.478)	0.225 (0.474)	0.226 (0.475)

Explained Variance (R^2^)	Level	Total	Level	Total	Level	Total
R^2^ Level-1	4.12%	0.88%	3.12%	0.65%	5.31%	1.10%
R^2^ Level-2	54.73%	43.02%	54.28%	42.94%	51.78%	41.06%
Total R^2^	43.89%		43.59%		42.17%	

^p < 0.1, ∗p < 0.05, ∗∗p < 0.0083 (corrected alpha for multiple comparisons – α/6), ∗∗∗p < 0.001.

For the association between sleep duration and the global performance score, no associations were found at either of the levels for the linear components of the model. For the quadratic terms, although no associations were found for the within-person component, there was a significant negative association at the between-person level (β_between-person quadratic_ = −0.080, SE = 0.018, p < 0.001, 95% CI: 0.116, −0.045). For the remaining two behavioral measures, positive associations with cognitive performance were found only at the between-person level for both step count (β_between-person_ = 0.104, SE = 0.031, p < 0.001, 95% CI: 0.042, 0.165) and screen time (β_between-person_ = 0.075, SE = 0.036, p = 0.038, 95% CI: 0.004, 0.146).

These between-person results (i.e., differences between people's means) suggest an inverted-U shape relationship between sleep duration and cognitive performance where, on average, people who typically have higher and lower mean sleep duration have lower global performance scores. Additionally, people who, on average, walk more and use their phones more have higher cognitive performance scores. At the within-person level (i.e., deviations from means), none of the digital markers of behavior showed associations with the performance scores. Overall, the effect size of the observed associations was small.

#### 3.2.2 Daily life behaviors and self-reported cognitive functioning

**Fig. 2:**
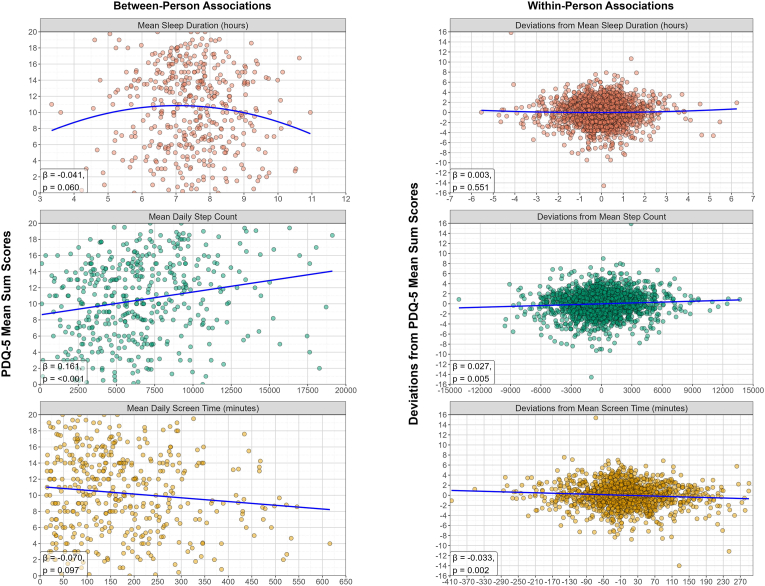
Panel plot illustrating the associations between digital markers of daily life behavior (sleep duration, step count and screen time) and smartphone-based assessments of self-reported cognitive functioning (i.e., PDQ-5 sum score). The left column contains the associations at the between-person level and includes participants' mean scores of the variables (n = 422 for sleep duration plot, n = 441 for step count plot, n = 419 for screen time plot). The right column contains the associations at the within-person level and includes the deviations from mean scores (t = 2064 for sleep duration plot, t = 2166 for step count plot, t = 1725 for screen time plot). Blue lines represent the average trend (quadratic for sleep duration, linear for step count and screen time). The standardized estimates (β) and significance (p) are shown at the bottom left corner of each plot. Results have been adjusted for age, gender and years of education.

As before, the three behavioral markers were separately modelled as predictors of self-reported cognitive functioning (i.e., PDQ-5 sum score). The null models yielded ICCs of 0.779-0.782, so between-person differences account for 78% of the variance in self-reported cognitive functioning, while the remaining 22% is due to within-person changes. Table 3 contains all standardized estimates and results are illustrated in Fig. 2. Unstandardized estimates to see relationships between variables in their original units are provided in the Appendix (section J).

**Table 3:** Standardized associations between digital markers of daily life behaviors (sleep duration, step count, and screen time) as predictors of self-reported cognitive functioning (PDQ-5 sum score). Level-1 is the within-person level. Level-2 is the between-person level. Significant associations are in bold. Abbreviations: N = unique participants, t = observations, PDQ-5 = 5-item Perceived Deficits Questionnaire.

Model (predictor – outcome)	Sleep duration – PDQ-5 sum score (N = 422, t = 2064)			Step count – PDQ-5 sum score (N = 441, 2166)			Screen time– PDQ-5 sum score (N = 419, t = 1725)		
Fixed effects coefficients	Estimate (SE)	95% CI	p value	Estimate (SE)	95% CI	p value	Estimate (SE)	95% CI	p value
Mean/Intercept	**−0.826 (0.223)**	**[-1.264, -0.388]**	**<0.001∗∗∗**	**−0.791 (0.201)**	**[-1.186, -0.397]**	**<0.001∗∗∗**	**−0.761 (0.211)**	**[-1.176, -0.345]**	**<0.001∗∗∗**
Time (relative to each person's start)	−4.6E-06 (6.3E-05)	[-1.3E-04, 1.2E-04]	0.942	−1.6E-05 (5.8E-05)	[-1.3E-04, 9.8E-05]	0.781	3.9E-05 (7.2E-05)	[-1.0E-04, 1.8E-04]	0.586
Behavior within-person component	0.002 (0.010)	[-0.017, 0.022]	0.807	**0.027 (0.010)**	**[0.008, 0.046]**	**0.005∗∗**	**−0.033 (0.011)**	**[-0.054, -0.012]**	**0.002∗∗**
Behavior within-person component (quadratic)	0.003 (0.005)	[-0.007, 0.013]	0.551	**-**	**-**	**-**		**-**	**-**
Behavior between-person component	0.009 (0.039)	[-0.069, 0.086]	0.822	**0.161 (0.037)**	**[0.087, 0.234]**	**<0.001∗∗∗**	−0.070 (0.042)	[-0.152, 0.013]	0.097 ^
Behavior between-person component (quadratic)	−0.041 (0.022)	[-0.084, 0.002]	0.060 ^	**-**	**-**	**-**		**-**	**--**
Age	**0.011 (0.003)**	**[0.005, 0.017]**	**<0.001∗∗∗**	**0.010 (0.003)**	**[0.005, 0.015]**	**<0.001∗∗∗**	**0.008 (0.003)**	**[0.003, 0.014]**	**0.004∗∗**
Gender (ref = male)	−0.161 (0.105)	[-0.367, 0.046]	0.127	−0.152 (0.093)	[-0.335, 0.032]	0.105	−0.089 (0.097)	[-0.280, 0.101]	0.359
Years of education	**0.026 (0.008)**	**[0.011, 0.042]**	**<0.001∗∗∗**	**0.025 (0.007)**	**[0.011, 0.040]**	**<0.001∗∗∗**	**0.026 (0.008)**	**[0.011, 0.041]**	**<0.001∗∗∗**

Random effects coefficients	Estimate (SD)	Estimate (SD)	Estimate (SD)
Level-2 error term	0.685 (0.828)	0.630 (0.794)	0.642 (0.801)
Level-1 error term	0.207 (0.455)	0.200 (0.448)	0.195 (0.442)

Explained Variance (R^2^)	Level	Total	Level	Total	Level	Total
R^2^ Level-1	0.05%	0.01%	0.43%	0.10%	0.57%	0.13%
R^2^ Level-2	7.79%	6.09%	11.28%	8.79%	6.97%	5.43%
Total R^2^	6.10%		8.89%		5.55%	

^p < 0.1, ∗p < 0.05, ∗∗p < 0.0083 (corrected alpha for multiple comparisons – α/6), ∗∗∗p < 0.001.

For the association between sleep duration and PDQ-5, no statistically significant associations were found at either of the levels, neither for the linear nor the quadratic components of the model. For step count, positive associations with the PDQ-5 sum score were found at both levels (β_between-person_ = 0.161, SE = 0.037, p < 0.001, 95% CI: 0.087, 0.234; β_within-person_ = 0.027, SE = 0.010, p = 0.005, 95% CI: 0.008, 0.046). Therefore, people who on average walked more tended to have higher PDQ-5 scores compared to those who walked less. Moreover, days when people had taken more steps than their average count were, on average, days when they also reported higher than average PDQ-5 scores. Finally, for screen time, negative associations were found only at the within-person level (β_within-person_ = −0.033, SE = 0.011, p = 0.002, 95% CI: 0.054, −0.012), indicating that days with higher than average screen time were linked to a worse than usual perception of one's own cognitive functioning. Similarly to the performance-based cognitive functioning models, effect sizes of the observed observation were small.

#### 3.2.3 Sensitivity analyses

In the sensitivity analysis including depression severity, because not all behavior-cognition pairs that were found in the main analysis had a PHQ-8 assessment that was temporally close, sample sizes were smaller (≈8% and 15% fewer participants and observations, respectively). Results showed that associations between daily life behaviors and self-reported cognitive functioning were no longer statistically significant, suggesting that variation in depression symptomatology and perceived cognitive functioning are partly shared. Associations with the performance-based global score remained statistically unchanged, indicating that those associations were independent of current depression severity (see Appendix, section H, with Supplementary Tables 1 and 2, for a discussion of these findings and more detailed results).

In the sensitivity analysis where cognitive assessments were linked to behavioral data from the week preceding them, the analysis yielded similar results for performance-based cognitive functioning, with between-person associations for sleep duration and step count remaining, while the between-person association with screen time was no longer observed. For self-reported cognitive functioning, the within-person association with step count disappeared, whereas negative associations with sleep duration (quadratic) and screen time emerged at the between-person level. Overall, results indicate that associations are timescale-dependent (see Appendix, section I, with Supplementary Tables 3 and 4, for a discussion of these findings and more detailed results).

Sample sizes were lower in both sensitivity analyses. Therefore, replication with larger samples would help confirm their robustness.

## 4 Discussion

This study explored how digital markers of daily life behaviors (sleep duration, step count, and smartphone screen time) relate to smartphone assessments of cognitive functioning (self-reported and performance-based) in individuals with recurrent MDD. We examined both between- and within-person associations using two days of behavioral data preceding each cognitive assessment. Our aim was to identify behavioral correlates of cognition in daily life, offering insights that could be used to support cognitive health in MDD. At the between-person level, all three behaviors were associated with performance-based cognition as expected, but only step count was positively linked to the self-reported measure. At the within-person level, none of the behaviors were associated with performance-based scores. Nonetheless, higher step count and lower screen time were associated with better self-reported cognitive functioning.

Sleep duration showed a non-linear association with performance-based cognition at the between-person level: both short and long average sleep durations were linked to lower performance. While we initially expected a positive linear relationship, this finding aligns with prior research on the impact of both sleep deprivation and oversleeping on cognition in mood disorders (Pearson et al., 2023). No association was found with self-reported cognition (PDQ-5), raising questions about whether individuals notice how sleep affects their perceived cognitive functioning. It may also suggest that performance-based tasks are more sensitive to detect the effect of sleep on cognition, at least with the two-day window we tested.

Step count emerged as the most consistent behavioral correlate. Between-person differences showed that individuals who walked more reported better cognitive functioning and performed better on tasks. At the within-person level, days with higher-than-usual step count were associated with better-than-usual PDQ-5 scores, suggesting that increases in daily steps may improve how people experience their cognitive functioning. However, this pattern did not extend to performance-based scores, indicating that PDQ-5 scores may be more sensitive to changes in average step count. These results suggest step count could be a valuable behavioral target. Although people with MDD often experience low energy and motivation, walking is simple to track, one of the most accessible and adaptable forms of physical activity (Rupp et al., 2024), and can be beneficial for cognitive health in depression (Cormack et al., 2024). However, translating these associations into interventions remains challenging, as interventions targeting step count still lack clear guidance regarding quantity, frequency, and context (Rupp et al., 2024). This is further complicated by evidence that shows Fitbit Charge 2 and 3 devices tend to overestimate step count (Bai et al., 2021; Hargis et al., 2018; Irwin and Gary, 2022).

Screen time revealed surprising results. At the between-person level, higher average screen time was linked to better performance scores, whereas within-person increases in smartphone use were associated with worse perceived cognitive functioning. One explanation is that between-person effects reflect mobile digital literacy or competence: individuals more familiar with smartphones may perform better on phone-based cognitive tasks due to, for example, better task switching capacity (Caton et al., 2022). Alternatively, certain types of engagement such as gaming (the tasks could be considered “mini-games”), may support cognitive functioning (Wilmer et al., 2017; Wilson et al., 2022). The negative within-person association with PDQ-5 may reflect the mental fatigue and distraction that comes with higher screen time (Dora et al., 2021) and/or the perception that high screen time in itself is problematic (Aalbers et al., 2022; Lanette et al., 2018). These findings raise questions about whether smartphone-based tasks actually measure cognitive performance or digital literacy, and about how to interpret performance data stemming from mobile devices.

Overall, we found divergences between the self-reported and performance-based measures. While the global performance score helped illustrate between-person differences, it may not fully reflect real-world cognitive functioning. More work is needed to understand whether mobile digital literacy inflates performance-based scores on smartphone tasks. Alternative performance measures such as keystroke typing behavior may offer complementary insights (Althoff et al., 2017; Ning et al., 2025). Moreover, despite our emphasis on within-person associations, none were found at this level. Perhaps performance on the THINC-it® tasks behaves more as a trait-like process, with ≈80% of the variance attributable to between-person differences. Another possibility is that low within-person variability in depressive symptoms may limit the presence of within-person variability in cognitive performance. In our sensitivity analysis, the ICC for depressive symptomatology was ≈0.70, indicating that most variance was between individuals (see Appendix, section H). Time of day effects, which were not explored here, may also be interesting to find within-person changes (Abdullah et al., 2016). Importantly, THINC-it® modules have only been validated in between-subject studies and in the clinic/laboratory. Thus, identifying smartphone-based tasks that are more sensitive to within-person variation in daily life settings, determining optimal assessment frequency and length, and clarifying which changes are clinically meaningful for depression will be critical for integrating performance-based cognitive measures into future digital remote patient monitoring studies (Daniëls et al., 2020; Mandryk et al., 2021; Moore et al., 2017; Sun et al., 2023). In contrast, the within-person associations observed with PDQ-5 suggest that self-reported measures may be better suited for remote monitoring of cognition in depression (Ross-Adelman et al., 2025).

An important limitation of this study involves the timing, quality, and availability of cognitive assessments. Although assessments were scheduled every six weeks, adherence varied. Some participants completed several in a short period, while others completed them inconsistently or with long missing gaps in between. Also, the THINC-it assessments were the least abundant out of all the available assessments in RADAR-MDD (Matcham et al., 2022a). These deviations from protocol affected data quality that raise broader questions about how to reliably collect cognitive data from individuals with depression, and about what motivates participation and engagement (de Angel et al., 2023; De Angel et al., 2022; Oetzmann et al., 2022; White et al., 2023). Additionally, our results are derived from a European population with recurrent MDD using Android phones and Fitbit devices, limiting generalizability. Relatedly, some associations differed when behaviors were summarized over one week versus a two-day window (see Appendix, section I). This suggests that the relationship between daily life behaviors and cognitive functioning can depend on the timescale we examine, which should be considered when posing research questions and comparing findings across studies. Still, a strength of this study lies in our effort to maximize use of available data through preprocessing, which nevertheless led to a large sample size for this clinical population.

## 5 Conclusions

This study contributes to a growing literature on the use of mobile technologies in depression, specifically focusing on cognition. It illustrates the importance of (a) distinguishing between self-reported and performance-based cognitive measures, (b) considering multiple daily life behaviors, and (c) separating between within- and between-person levels, as results differ per behavior-cognitive outcome pair and per level. For performance-based measures, key challenges include identifying tasks sensitive to within-person change, determining optimal assessment frequency, and understanding which changes hold clinical significance for depression. For self-reported measures, repeated assessment shows promise for remote patient monitoring. In conclusion, our findings offer insight into how everyday behaviors relate to cognitive functioning in depression. Although our results are correlational and should not be interpreted causally, they highlight the potential wearables and smartphones have to capture meaningful information that can be used to support cognitive health in individuals with depression.

## Data sharing

The data needed to replicate the analyses in this paper are not publicly accessible. Nonetheless, the code required for replication, alongside supplementary materials, are available on the OSF project URL (https://osf.io/r36mk/).

## Declarations of generative AI and AI-assisted technologies in the manuscript preparation process

During the preparation of this work the corresponding author used Open AI's ChatGPT 4.0 to bring conciseness to the manuscript and debug code when needed. After using this tool, the corresponding author reviewed and edited the content as needed and takes full responsibility for the content of the published article.

## Funding

This work is funded by Stress in Action and done in collaboration with the RADAR-CNS Consortium. The research project “Stress in Action” (www.stress-in-action.nl) is financially supported by the 10.13039/501100003246Dutch Research Council and the Dutch Ministry of Education, Culture and Science (NWO gravitation [grant number 024.005.010]). The RADAR-CNS project received funding from the “10.13039/501100010767Innovative Medicines Initiative 2 Joint Undertaking” [grant agreement number 115902]. This Joint Undertaking received support from the European Union' s Horizon 2020 Research and Innovation Program and the 10.13039/100013322EFPIA (www.imi.europa.eu). This work was also funded by the 10.13039/501100004837Spanish Ministry of Science and Innovation [grant number TED2021-131106 B-I00], by the European Social Fund (EU), and by the Aragón Government (Spain) through BSICoS group [grant number T39_23R]. The funding bodies have not been involved in the study design, data collection, data analysis, data interpretation, or writing of the report.

## Declaration of competing interest

The authors declare the following financial interests/personal relationships which may be considered as potential competing interests: Peter Annas and Maria Dalby were full-time employees of H. Lundbeck A/S at the time of data collection. No other co-authors have conflicts of interests to declare.
