# Supplementary material for: Sleep, Steps, and Screens: Between- and within-person effects of digital markers of daily life behaviors on smartphone-based assessments of cognitive functioning in depression
Source: Neurosci Appl. 2026 Feb 14;5:106985. doi: 10.1016/j.nsa.2026.106985 (PMC12936773; doi:10.1016/j.nsa.2026.106985)
Supplement: Multimedia component 1 [file mmc1.docx]

**SUPPLEMENTARY MATERIALS (APPENDIX) FOR:**

**Sleep, Steps, Screens: Between and within-person effects of Digital Markers of Daily Life Behaviors on Smartphone-based assessments of Cognitive Functioning in Depression**

**Marcos-Ross Adelman (**[**marcos.ross@amsterdamumc.nl**](mailto:marcos.ross@amsterdamumc.nl)**)**

In this file you will find supplementary figures and tables referred to in the main manuscript, and explanations on data preprocessing and sensitivity analysis decisions.

Outline

1. Supplementary Figure 1. Correlation between THINC-it^®^ measures.
2. Supplementary Figure 2. THINC-it^®^ completion throughout the day
3. Supplementary Figures 3 and 4. Sensitivity and internal consistency/reliability of THINC-it^®^ measures.
4. Details on adherence and data availability.
5. Handling of outliers during data-preprocessing.
6. Supplementary Figure 5. Flowchart of sample selection used for this study.
7. Supplementary Figures 6 and 7. Plots of variables over time.
8. Supplementary Tables 1 and 2. Sensitivity analysis. Including depression severity as a predictor.
9. Supplementary Tables 3 and 4. Sensitivity analysis. Using a one-week window instead of a two-day window.
10. Reporting the unstandardized coefficients for a more tangible interpretation.
11. References.
12.
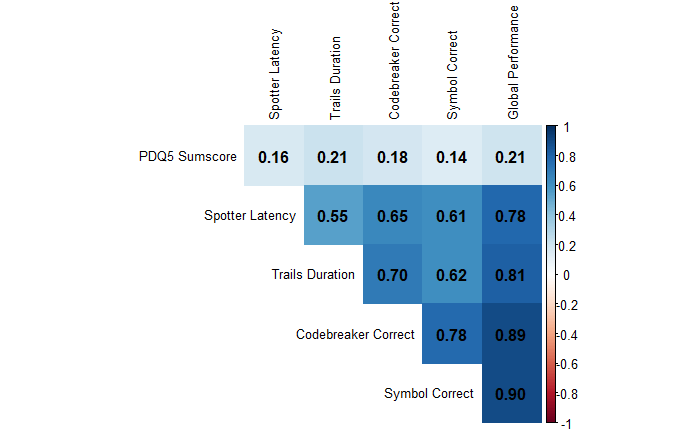
**Correlation between THINC-it^®^ measures**

**Supplementary Figure 1.** Correlation matrix between THINC-it^®^ modules, including the self-reported measure (PDQ-5), the four performance tasks (Spotter, Codebreaker, Symbol Check, Trails) and the normalized 0-100 global performance score.

Notes on THINC-it^®^ measures:

- The fact that self-reported and performance-based measures of cognitive functioning independently contribute to patient functioning drove our decision to use them as separate outcomes in the analysis (McIntyre et al., 2017). The low correlation between them strengthens it.
- The correlations we found between the individual task scores, as well as the desire to avoid multiple testing, motivated the decision to create a normalized global performance score.
- The global performance score was created whenever a participant completed two or more tasks at any given measurement occasion. For occasions when participants completed only one task, their normalized score for that task was used as their global score. To clarify, on 87.70% of occasions (n = 2678), all 4 tasks were available. On 6.52% of occasions (n = 199), 3 tasks were available. On 0.88% of occasions (n = 27), 2 tasks were available. Only on 4.90% of observations (n = 148) was there only 1 task available.

1. **THINC-it^®^ completion throughout the day**


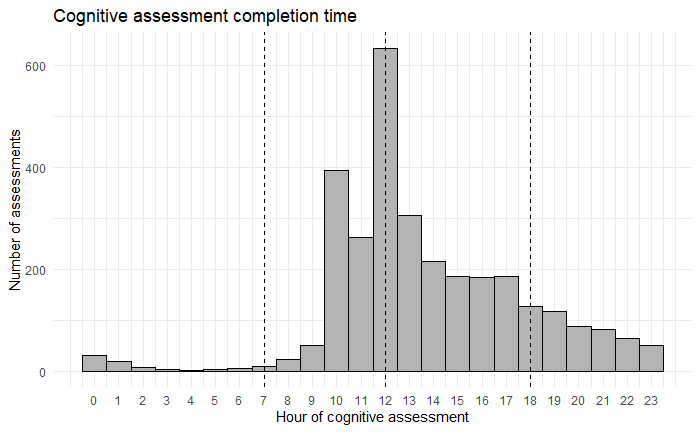


**Supplementary Figure 2.** Histogram indicating when THINC-it^®^ assessments were completed throughout the day. Dotted lines delineate early-morning hours (00:00–06:59), morning (7:00-11:59), afternoon (12:00-17:59), and evening (18:00-23:59). They were most frequently completed in the afternoon (56.1%), followed by the morning (24.2%) and evening (17.4%), A small proportion of assessments (2.3%) were completed during late-night and early-morning hours.

1. **Sensitivity and internal consistency/reliability of THINC-it^®^ measures**

For a questionnaire or task to be useful in studying a construct of interest, it must be able to capture sufficient variance (i.e., be sensitive to change). In this study, we used composite measures of performance-based and self-reported cognitive functioning that contained sub-components. The global cognitive performance score summarizes four tasks assessing distinct cognitive domains: attention (Spotter), working memory (Symbol Check), processing speed (Codebreaker), and attention switching (Trails). PDQ-5 is a shortened version of the PDQ-20 that has 5 items that address different domains of perceived cognitive functioning: attention/concentration, retrospective memory, prospective memory, and planning/organization (National Multiple Sclerosis Society, 2025, pages 27-28 of the attached manual).

To evaluate the presence of floor and ceiling effects for the individual and global components (i.e., their sensitivity to detect change), we plotted histograms to visually check the distribution of the items and estimated its skewness. No floor or ceiling effects were found for any of the individual tasks of the global performance score nor for the individual items of the PDQ-5 (see below pages 5 and 6 for the plots).

Furthermore, because both composites are multidimensional, combining individual components that assess different dimensions of cognition, we tested their internal consistency/reliability using McDonald’s omega (Kalkbrenner, 2023). For the composite measures to be a reliable way to measure the construct of interest (global cognitive performance or perceived cognitive functioning), their individual components must have consistency among them. Said differently, they must measure the same thing (to a certain degree) that the composite score represents. Specifically, hierarchical omega (ω_h_) was used to assess the internal consistency:

- ω_h_ for the global performance score = 0.809
- ω_h_ for PDQ-5 = 0.797

These scores suggest that the majority of variance in the composite cognitive measures we use (around 80%) is attributable to a shared latent factor, with a smaller proportion reflecting task/question-specific variance. These findings support the internal consistency of both composite measures.

**Supplementary Figure 3.** Histograms of each of the THINC-it^®^ tasks (in grey) and of the global cognitive performance score (in blue) to check for floor or ceiling effects. The ‘Trails’ task shows the largest skewness. That may be due to the unexpectedly long completion times, rather than actual floor effects.


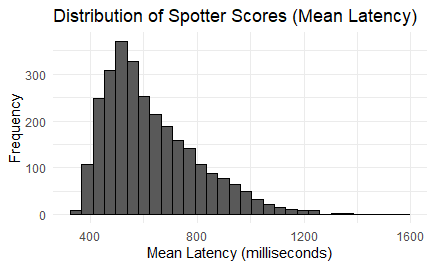

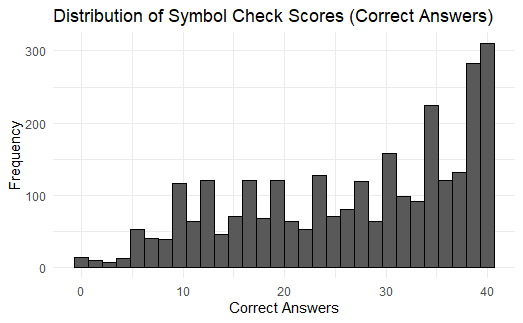

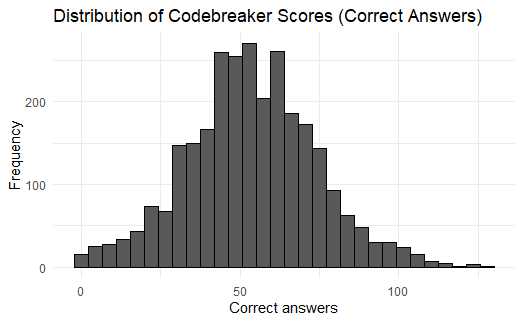

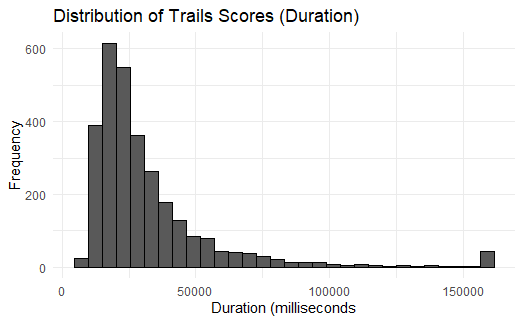

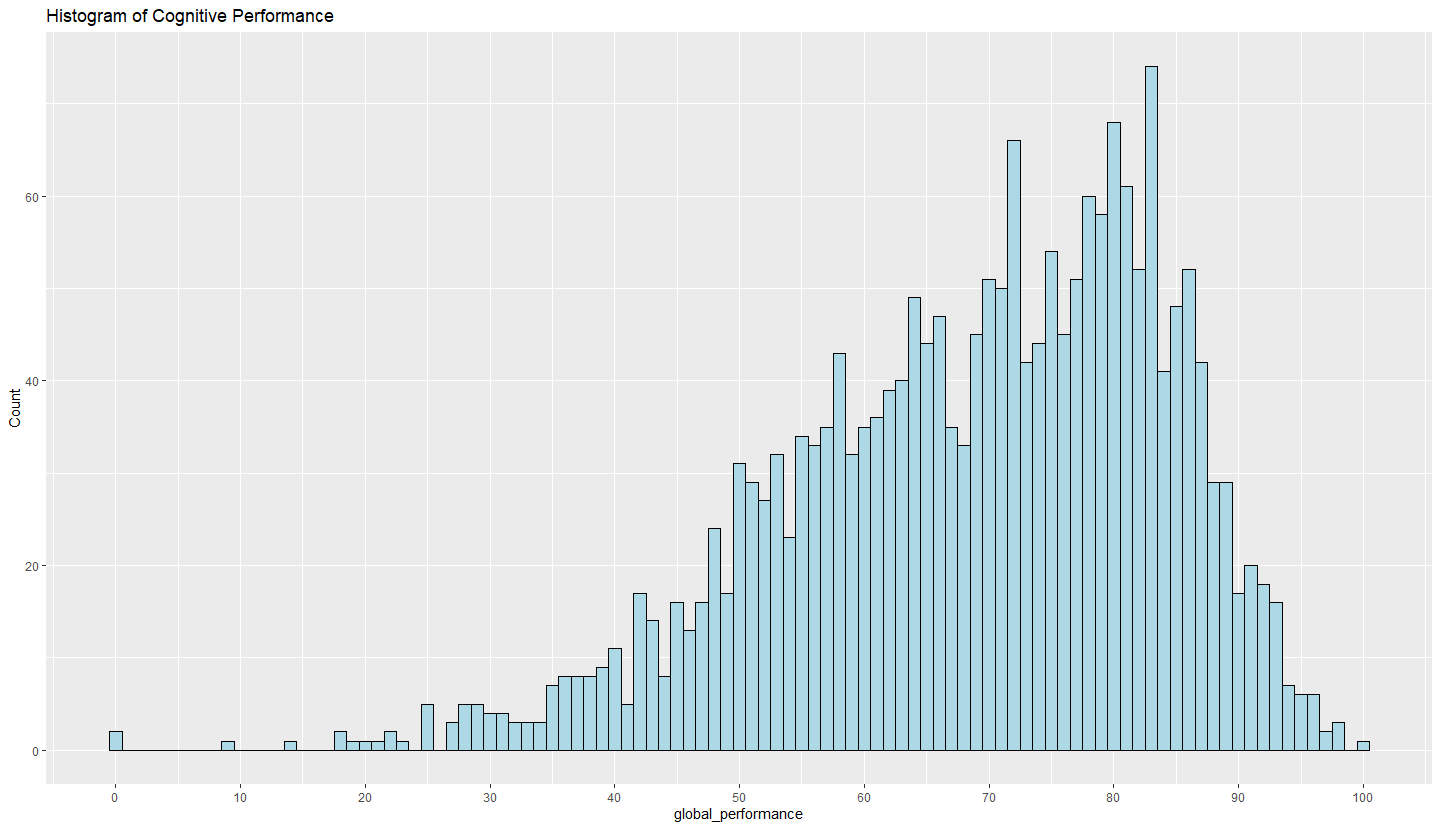


**
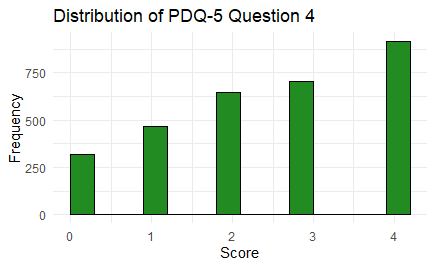

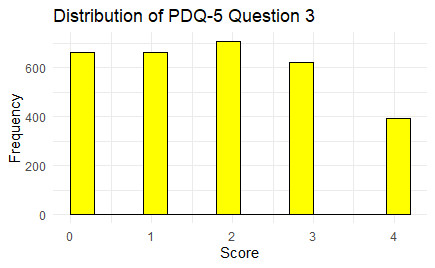

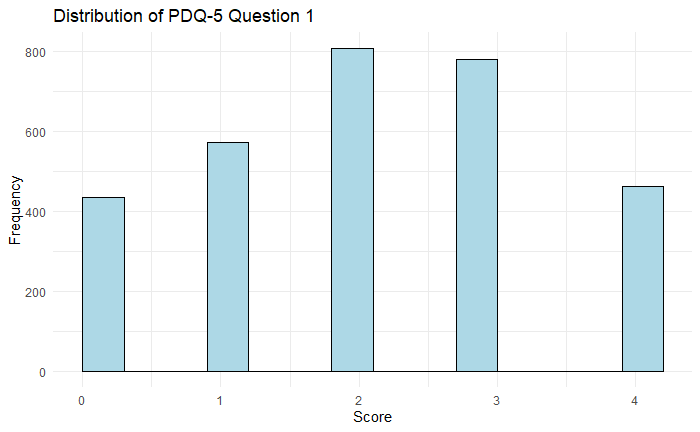

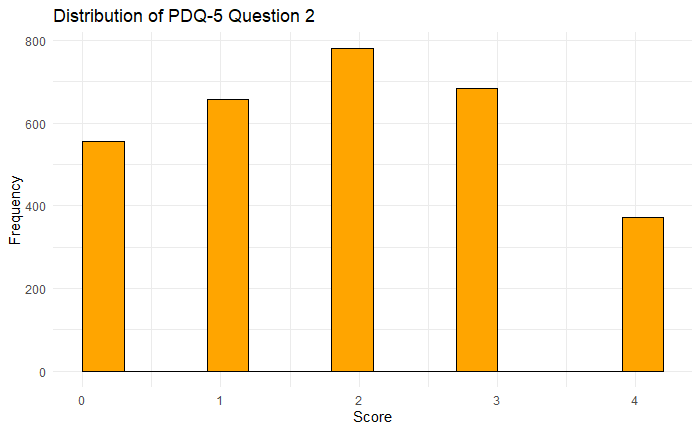
**

**
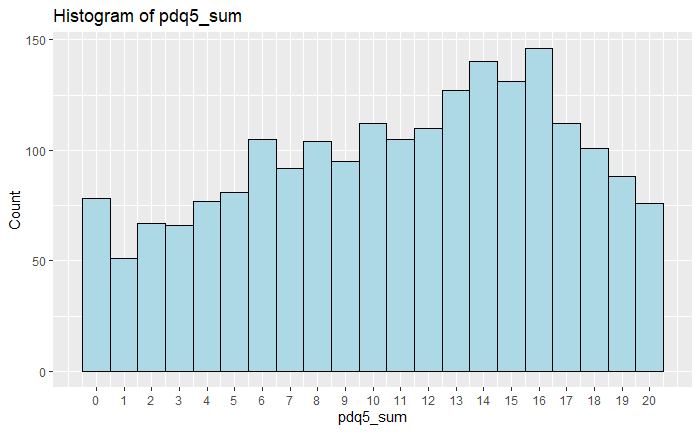
**

**
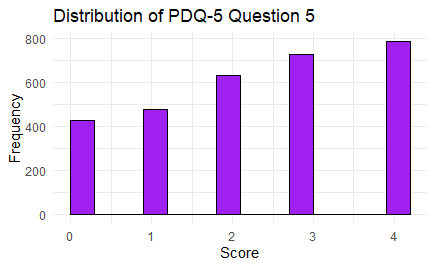
**

**Supplementary Figure 4.** Histograms of each of the PDQ-5 questions (left column and two top right plots) and the sum score (bottom right plot) to check for floor or ceiling effects.

1. **Details on adherence and data availability**

This section contains information on adherence to protocol and data availability before merging the cognitive assessments with the behavioral data. Everything was calculated within participants and then summed or averaged across the sample, using each participant’s first observed assessment as the reference point.

Beginning with THINC-it® data, adherence to the intended six-week schedule was operationalized as the spacing between consecutive completed assessments. Overall, there was substantial irregularity and, thus, deviations from protocol. Around 15.5% of assessments occurred on the same day as a prior assessment, while an additional 7.5% occurred on the following day. Another 9% of assessments were provided within the same week (2-7 days after the previous assessment), and 17% were provided between 8-38 days after the previous one. Only 32.5% of assessments were given between 38-46 days after a previous one (i.e., around the intended schedule). Finally, 18.5% of assessments were spaced more than 46 days apart. This irregularity motivated the decision to keep assessments that were at least one week apart to maximize data availability and avoid using measurements that were too close together. This led to the 3050 PDQ-5 and 3052 global cognitive performance assessments that were on average 80 days apart (SD = 86, median = 69 days), as reported in the main manuscript.

Continuing with the passive data streams, in the main manuscript we report the total number of recorded days per behavior and mean recorded days per participant. What follows is more details on adherence. Because participants contributed data over differing periods of time (i.e., different start and end dates), adherence to protocol was operationalized as data availability. We calculated the percentage of days with recorded data relative to each person’s time in study (i.e., how many days was a person in the study, how many of those days did they wear their Fitbit and thus followed protocol and provided data?). For sleep duration, mean percentage of days with recorded data was 62.4% (SD = 23.3, median = 68, range: 1.1 – 100%). For step count, the mean percentage of days with recorded data was 74.2% (SD = 23.6, median = 81.6, range: 2.7 – 100%). For screen time data, mean percentage of days with recorded data was 65.9% (SD = 31.4, median = 75.6%, range: 0.7 – 100%).

Code that was used to calculate these results can be found in the OSF project URL under ‘Code → 1. Data Preprocessing ([https://osf.io/r36mk/)](https://osf.io/r36mk/). There is one R markdown file per variable.

For your information, the above mentioned findings pertain to the RADAR-MDD subsample used in our study, whereas Matcham et al. (2022) provide information for the full RADAR-MDD sample.

1. **Handling of outliers during data preprocessing**

We addressed outliers using domain knowledge and winsorization, consistently applied at ±3 standard deviations from the mean (Kwak & Kim, 2017):

- For the *Codebreaker* task we found negative scores (n = 69), which is impossible considering the task measures correct answers.
- For the *Trails* task we found impossibly long scores (e.g., over 9 minutes completion time), so we winsorized the upper end at +3 standard deviations from the mean (n = 79). The new upper cap became 2.65 minutes.
- For sleep duration, extreme scores were found (a maximum value of 21.2 hours and a minimum of 0.69 hours). Thus, we winsorized the both ends at ± 3 standard deviations from the mean (n = 5 capped observations on the low end, n = 765 capped observations on the high end). The new range became 2 – 13 hours of sleep duration.
- For screen time (smartphone unlock duration), we found extremely high values such as 21 hours. Thus, we winsorized the upped end at +3 standard deviations from the mean (n = 1958). The new upper cap became 634 minutes, around 10.5 hours.
- For step count we found minimum and maximum values ranging from 0 to 83002 steps (around 60-65 km), both impossible. Thus, days with less than 100 steps were dropped (n = 110 959), and high values were capped at 30000 steps (n = 289), following what previous research with step count has done (Vos et al., 2025). Moreover, RADAR-MDD had a quality metric for recorded step count days that assessed the percentage of data that was missing within a day. So, based on prior work, days with less than 16 hours of device wear (Difrancesco et al., 2019) were dropped (n = 1082).
  - A note on this, if you are wondering why this quality control metric was not applied for sleep duration and screen time days, it is because it was not present with these behavioral measures.

All preprocessing code (not just handling outliers) is available for replication in the OSF project URL under ‘Code → 1. Data Preprocessing’. There you will find code that was used to preprocess each of the variables used in our study (<https://osf.io/r36mk/>).

1. **Flowchart of sample selection**

Samples after quality control (e.g., duplicates, impossible scores, outliers)

Initial RADAR-MDD sample:

N = 623

Self-reported cognitive functioning (PDQ-5)

(assessed every 6 weeks):

N = 523

t = 4172

Performance-based cognitive functioning

(assessed every 6 weeks):

Daily life behaviors

(assessed over 24h periods, daily):

Symbol Check (working memory):

N = 499

t = 3897

Spotter (attention):

N = 492

t = 3764

Step count:

N = 574

t = 209 789

Sleep duration:

N = 578

t = 158 632

Screen time:

N = 589

t = 154 129

Sample that provided PDQ-5 assessments that were at least one week apart:

N = 523

t = 3050

Trails

(attention switching):

N = 521

t = 4085

Codebreaker (processing speed):

N = 498

t = 3787

Sample who completed at least one performance-based measure with assessments that were at least one week apart:

N = 521

t = 3052

Samples that included all cognitive assessments that could be linked to behavioral data from the day of- and the day preceding the cognitive assessment date:

N = 502

Performance + Step count:

N = 439

t = 2162

Performance + Phone use:

N = 418

t = 1729

Performance + Sleep duration:

N = 422

t = 2063

PDQ-5 + Step count:

N = 441

t = 2166

PDQ-5 + Screen time:

N = 419

t = 1725

PDQ-5 + Sleep duration:

N = 422

t = 2064

**Supplementary Figure 5**. Flowchart of sample selection for all behavioral and cognitive functioning measure combinations for main analysis. Note: most participants completed all four performance tasks per measurement occasion. Approximately 88% of observations (t = 2678) were thus complete assessments. Abbreviations: N = participants, t = observations, PDQ-5 = 5-item Perceived Deficit Questionnaire.

1. **Plots of variables over time**


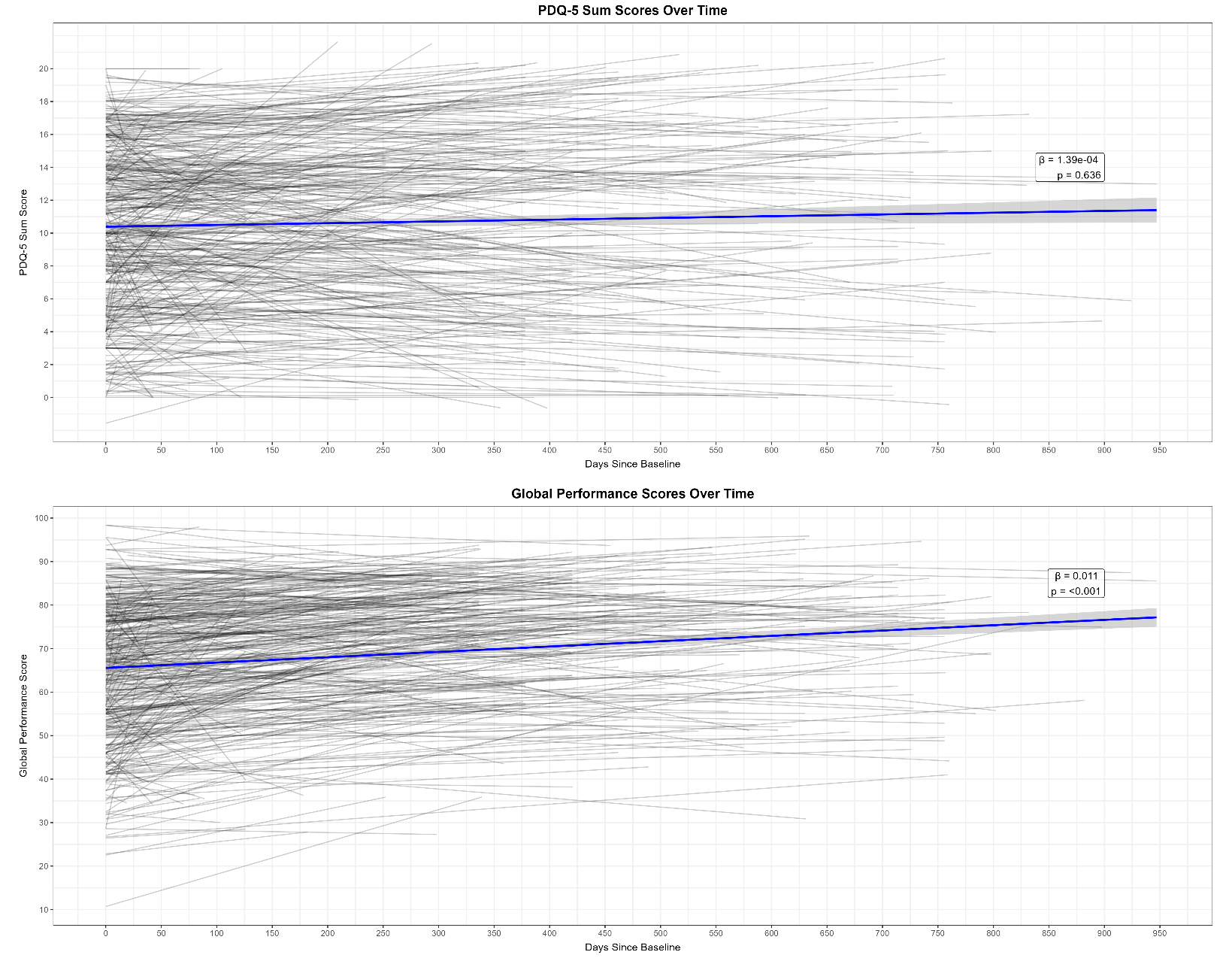


**Supplementary Figure 6.** Time series plots of the cognitive functioning measures (PDQ-5 at the top and global performance scores at the bottom). Time is calculated as “days since baseline”, which is relative to each participant’s starting date. Gray lines represent each individual trajectory. Blue lines represent the average linear trend of the whole sample’s trajectory, with the effect of time shown by the β and p-values in each plot.


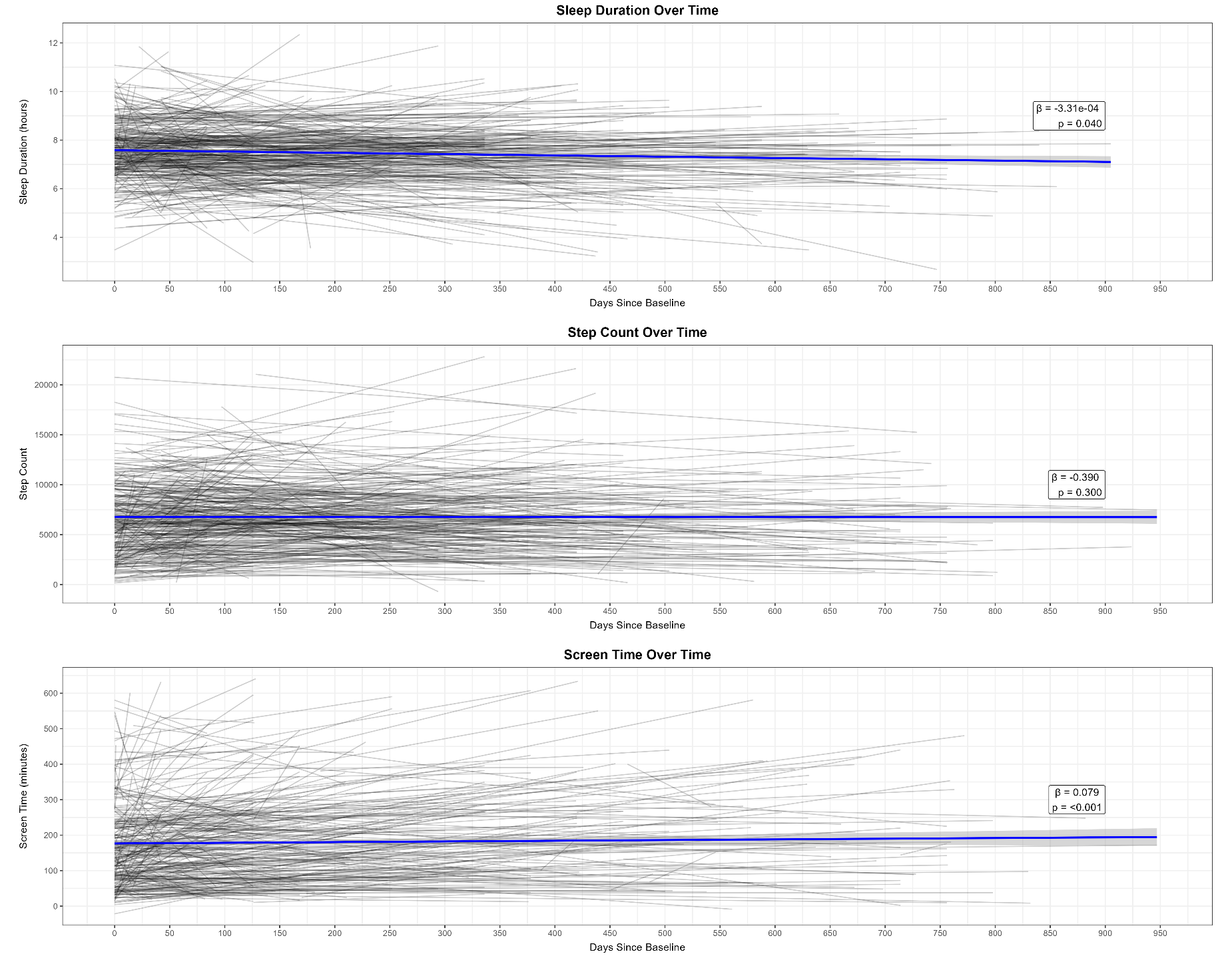


**Supplementary Figure 7.** Time series plots of the behavioral measures (sleep duration at the top, step count in the middle, and screen time at the bottom). Time is calculated as “days since baseline”, which is relative to each participant’s starting date. Gray lines represent each individual trajectory. Blue lines represent the average linear trend of the whole sample’s trajectory, with the effect of time shown by the β and p-values in each plot.

1. **Sensitivity analysis. Including depression severity as a predictor.**

Given the associations found between cognitive functioning and depression severity found in prior work (Ross-Adelman et al., 2025), we tested whether the observed associations were independent from current depression levels. To this end, we ran a sensitivity analysis including depression severity scores that were temporally aligned with each behavioral predictor-cognitive outcome pair. Depression severity was measured with the 8-item Patient Health Questionnaire (PHQ-8; Kroenke et al., 2009). Items were summed, resulting in a composite score ranging from 0 to 24 where higher scores reflect greater severity.

Temporal alignment meant finding PHQ-8 assessments that were within a ±5 day range from any given behavior-cognitive outcome pair and linking them to said pair. Not all behavior-cognition pairs that were found in the main analysis had a PHQ-8 assessment that was temporally close for the sensitivity analysis. Thus, sample sizes were slightly lower (around 15% fewer observations, and 8% fewer participants), so caution should be taken when interpreting the findings. Results are illustrated below in Supplementary Table 1 (with global performance score as outcome) and Supplementary Table 2 (with PDQ-5 as outcome).

PHQ-8 scores were negatively associated with both the performance-based cognitive measure (between-person: β = -0.206, SE = 0.034, p<0.001, 95% CI: -0.274, -0.138; within-person: β = -0.031, SE = 0.011, p = 0.004, 95% CI: -0.052, -0.010), and the self-reported measure (between-person: β = -0.642, SE = 0.031, p<0.001, 95% CI: -0.704, -0.580; within-person: β = -0.214, SE = 0.009, p<0.001, 95% CI: -0.232, -0.196). Including PHQ-8 did not change the associations found between daily behaviors and the performance-based scores, whereas associations with self-reported cognitive functioning were no longer statistically significant.

To contextualize these findings, especially the within-person associations, we also estimated the intraclass correlation coefficients (ICCs) for depression severity using null models with PHQ-8 scores as the outcome. Because changes in cognitive functioning are part of depression symptomatology, within-person associations between the daily behaviors and cognitive functioning rely on the presence of sufficient variance of PHQ-8 scores at the within-person level. The ICC for PHQ-8 across models ranged between 0.688-0.706, indicating that approximately 70% of the variance is located at the between-person level, and 30% is at the within-person level.

For performance-based cognitive functioning, the fact that the between-person behavior-cognition associations found in the main analysis remain after including PHQ-8 suggests they are independent from current depression severity. At the within-person level, the absence of associations may be explained by the fact that the largest chunk of PHQ-8 variance is at the between-person level (approximately 2/3 of all variance). Said differently, without enough within-person symptomatology variance, it may be hard to find within-person variance in cognitive performance.

For self-reported cognitive functioning, the disappearance of both the between- and within-person associations found in the main analysis suggest that our findings are not independent from current depression severity. This makes sense because, as mentioned above, variation in perceived cognitive functioning and in depressive symptomatology are partly shared. Nevertheless, the main analysis remains informative because it illustrates how everyday behaviors co-vary with self-reported cognitive functioning in the context of recurrent MDD.

**Supplementary Table 1.** Standardized associations between digital markers of daily life behaviors (sleep duration, step count, and screen time) and performance-based cognitive smartphone assessments (global performance score of THINC-it^®^ tasks), including depression severity (PHQ-8 sum score) in the model as a sensitivity analysis. Level-1 is the within-person level. Level-2 is the between-person level. Significant associations are in bold. Abbreviations: N = unique participants, t = observations.

| **Model (Predictor – Outcome)**  **Sensitivity analysis: with PHQ-8 in model** | **Sleep duration – Global Performance score**  **(N = 392, t = 1783)** | | | | **Step count – Global Performance score**  **(N = 403, t = 1834)** | | | | **Screen time – Global Performance score**  **(N = 388, t = 1453)** | | | |
| --- | --- | --- | --- | --- | --- | --- | --- | --- | --- | --- | --- | --- |
| **Fixed effects coefficients** | **Estimate (SE)** | **95% CI** | | **p value** | **Estimate (SE)** | **95% CI** | | **p value** | **Estimate (SE)** | **95% CI** | | **p value** |
| Mean/Intercept | **1.471 (0.180)** | **[1.117, 1.826]** | | **<0.001***** | **1.351 (0.169)** | **[1.019, 1.684]** | | **<0.001***** | **1.265 (0.182)** | **[0.908, 1.622]** | | **<0.001***** |
| Time (relative to each person’s start) | **8.72E-04 (7.14E-05)** | **[7.32E-04,1.01E-03]** | | **<0.001***** | **7.35E-04 (6.53E-05)** | **[6.07E-04, 8.63E-04]** | | **<0.001***** | **9.02E-04 (8.43E-05)** | **[7.36E-04, 1.07E-03]** | | **<0.001***** |
| Behavior within-person component | 0.005 (0.011) | [-0.016, 0.026] | | 0.634 | 0.005 (0.010) | [-0.015, 0.026] | | 0.615 | 0.022 (0.012) | [-0.001, 0.045] | | 0.062^ |
| Behavior within-person component (quadratic) | -0.007 (0.005) | [-0.018, 0.003] | | 0.171 | **-** | **-** | | **-** | **-** | **-** | | **-** |
| Behavior between-person component | 0.009 (0.032) | [-0.053, 0.071] | | 0.779 | **0.077 (0.033)** | **[0.012, 0.142]** | | **0.020*** | **0.089 (0.037)** | **[0.017, 0.161]** | | **0.016*** |
| Behavior between-person component (quadratic) | **-0.063 (0.017)** | **[-0.097, -0.029]** | | **<0.001***** | **-** | **-** | | **-** | **-** | **-** | | **-** |
| PHQ-8 within-person component | **-0.031 (0.011)** | **[-0.052, -0.010]** | | **0.004**** | **-0.034 (0.010)** | **[-0.054, -0.014]** | | **0.001**** | -0.017 (0.012) | [-0.040, 0.006] | | 0.145 |
| PHQ-8 between-person component | **-0.206 (0.034)** | **[-0.274, -0.138]** | | **<0.001***** | **-0.227 (0.035)** | **[-0.295, -0.159]** | | **<0.001***** | **-0.240 (0.036)** | **[-0.311, -0.169]** | | **<0.001***** |
| Age | **-0.043 (0.002)** | **[-0.048, -0.039]** | | **<0.001***** | **-0.043 (0.002)** | **[-0.047, -0.039]** | | **<0.001***** | **-0.042 (0.002)** | **[-0.046, -0.037]** | | **<0.001***** |
| Gender (ref=male) | -0.029 (0.083) | [-0.192, 0.133] | | 0.725 | 0.022 (0.078) | [-0.131, 0.175] | | 0.776 | -0.059 (0.084) | [-0.224, 0.105] | | 0.478 |
| Years of education | **0.025 (0.007)** | **[0.013, 0.038]** | | **<0.001***** | **0.028 (0.006)** | **[0.016, 0.040]** | | **<0.001***** | **0.032 (0.007)** | **[0.019, 0.045]** | | **<0.001***** |
| **Random effects coefficients** | **Estimate (SD)** | | | | **Estimate (SD)** | | | | **Estimate (SD)** | | | |
| Level-2 error term | 0.367 (0.606) | | | | 0.365 (0.604) | | | | 0.403 (0.635) | | | |
| Level-1 error term | 0.203 (0.451) | | | | 0.196 (0.443) | | | | 0.200 (0.447) | | | |
| **Explained Variance (R^2^)** | **Level** | | **Total** | | **Level** | | **Total** | | **Level** | | **Total** | |
| R^2^ Level-1 | 7.77% | | 1.50% | | 6.72% | | 1.25% | | 8.26% | | 1.54% | |
| R^2^ Level-2 | 60.36% | | 48.71% | | 60.19% | | 49.00% | | 58.59% | | 47.69% | |
| Total R^2^ | 50.21% | | | | 50.25% | | | | 49.23% | | | |

^p<0.1, *p<0.05, **p<0.0083 (corrected alpha for multiple comparisons – α/6), ***p<0.001

**Supplementary Table 2.** Standardized associations between digital markers of daily life behaviors (sleep duration, step count, and screen time) and self-reported cognitive functioning (PDQ-5 sum score), including depression severity (PHQ-8 sum score) in the model as a sensitivity analysis. Level-1 is the within-person level. Level-2 is the between-person level. Significant associations are in bold. Abbreviations: N = unique participants, t = observations, PDQ-5 = 5-item Perceived Deficits Questionnaire

| **Model (Predictor – Outcome)**  **Sensitivity analysis: with PHQ-8 in model** | **Sleep duration – PDQ-5 sum score**  **(N = 392, t = 1783)** | | | | **Step count – PDQ-5 sum score**  **(N = 403, t = 1834)** | | | | **Screen time – PDQ-5 sum score**  **(N = 388, t = 1451)** | | | |
| --- | --- | --- | --- | --- | --- | --- | --- | --- | --- | --- | --- | --- |
| **Fixed effects coefficients** | **Estimate (SE)** | **95% CI** | | **p value** | **Estimate (SE)** | **95% CI** | | **p value** | **Estimate (SE)** | **95% CI** | | **p value** |
| Mean/Intercept | -0.201 (0.165) | [-0.526, 0.124] | | 0.225 | -0.161 (0.152) | [-0.459, 0.137] | | 0.290 | -0.121 (0.157) | [-0.429, 0.187] | | 0.441 |
| Time (relative to each person’s start) | 1.07E-04 (6.17E-05) | [-1.37E-05, 2.29E-04] | | 0.082^ | 8.78E-05 (5.65E-05) | [-2.30E-05, 1.99E-04] | | 0.120 | -1.33E-05 (7.19E-05) | [-1.54E-04, 1.28E-04] | | 0.853 |
| Behavior within-person component | -0.007 (0.009) | [-0.025, 0.011] | | 0.461 | 0.009 (0.009) | [-0.008, 0.027] | | 0.311 | -0.017 (0.010) | [-0.037, 0.002] | | 0.085^ |
| Behavior within-person component (quadratic) | 0.006 (0.005) | [-0.003, 0.015] | | 0.191 | **-** | **-** | | **-** | **-** | **-** | | **-** |
| Behavior between-person component | 0.021 (0.029) | [-0.036, 0.078] | | 0.474 | 0.058 (0.030) | [-3.04E-05, 0.116] | | 0.050^ | -0.057 (0.032) | [-0.119, 0.005] | | 0.073^ |
| Behavior between-person component (quadratic) | -0.015 (0.016) | [-0.046, 0.016] | | 0.332 |  |  | |  | **-** | **-** | | **-** |
| PHQ-8 within-person component | **-0.214 (0.009)** | **[-0.232, -0.196]** | | **<0.001***** | **-0.209 (0.009)** | **[-0.227, -0.192]** | | **<0.001***** | **-0.189 (0.010)** | **[-0.208, -0.169]** | | **<0.001***** |
| PHQ-8 between-person component | **-0.642 (0.031)** | **[-0.704, -0.580]** | | **<0.001***** | **-0.622 (0.031)** | **[-0.683, -0.562]** | | **<0.001***** | **-0.625 (0.031)** | **[-0.686, -0.563]** | | **<0.001***** |
| Age | 0.003 (0.002) | [-0.001, 0.008] | | 0.096^ | 0.003 (0.002) | [-0.001, 0.007] | | 0.153 | 0.002 (0.002) | [-0.002, 0.006] | | 0.417 |
| Gender (ref=male) | -0.065 (0.076) | [-0.214, 0.084] | | 0.394 | -0.104 (0.070) | [-0.242, 0.033] | | 0.137 | -0.133 (0.072) | [-0.275, 0.009] | | 0.066^ |
| Years of education | 0.004 (0.006) | [-0.007, 0.016] | | 0.462 | 0.005 (0.006) | [-0.006, 0.016] | | 0.383 | 0.008 (0.006) | [-0.003, 0.020] | | 0.162 |
| **Random effects coefficients** | **Estimate (SD)** | | | | **Estimate (SD)** | | | | **Estimate (SD)** | | | |
| Level-2 error term | 0.315 (0.561) | | | | 0.297 (0.545) | | | | 0.302 (0.549) | | | |
| Level-1 error term | 0.151 (0.389) | | | | 0.146 (0.382) | | | | 0.145 (0.380) | | | |
| **Explained Variance (R^2^)** | **Level** | | **Total** | | **Level** | | **Total** | | **Level** | | **Total** | |
| R^2^ Level-1 | 28.20% | | 6.18% | | 27.21% | | 6.07% | | 25.35% | | 5.50% | |
| R^2^ Level-2 | 57.94% | | 45.25% | | 58.82% | | 45.93% | | 56.99% | | 44.62% | |
| Total R^2^ | 51.43% | | | | 52.00% | | | | 50.13% | | | |

^p<0.1, *p<0.05, **p<0.0083 (corrected alpha for multiple comparisons – α/6), ***p<0.001

1. **Sensitivity analysis. Using a one-week window instead of a two-day window.**

To assess the stability of our findings, we examined whether associations differed when behavioral data were aggregated over a one-week window (i.e., data from the day of and six days preceding the cognitive assessment) rather than the two-day window used in the main analyses. Conceptually speaking, averaging data over longer time smooths day-to-day fluctuations, reducing sensitivity to short-lived effects while emphasizing habitual or cumulative behavioral patterns.

Behavioral measures were linked to a cognitive assessment only if at least 4 out of 7 days contained data (Sun et al., 2023). This led to smaller sample sizes (i.e., approximately 9-20% fewer participants and 11-19% fewer observations depending on the dataset). Results are shown in Supplementary Table 3 (with global performance score as outcome) and Supplementary Table 4 (with PDQ-5 as outcome).

The correlation between the behavioral data from both time windows were quite high (0.775 for sleep duration, 0.863 for step count, and 0.925 for screen time) and ICCs for the global performance score and PDQ-5 remained the same around 0.80.

Overall, results based on a one-week window of behavioral data were largely consistent for performance-based cognitive functioning, where people with lower mean step count (β between-person = 0.099, SE = 0.034, p = 0.004, 95% CI: 0.032, 0.167) and people who have higher and lower than the mean sleep duration (βbetween-person quadratic = -0.073, SE = 0.022, p <0.001, 95% CI: -0.116, -0.030) score lower in the global performance score. In contrast, the between-person association with screen time observed in the main analysis was no longer present. This suggests that the relationship we found between phone use and task performance may operate on shorter timescales, but not with more habitual screen exposure.

For self-reported cognitive functioning, extending the temporal window showed that PDQ-5 may be more sensitive to habitual behavioral patterns because between-person associations that were not found in the main analysis were found here. Specifically, sleep duration showed a negative quadratic association (βbetween-person quadratic = -0.058, SE = 0.027, p = 0.030, 95% CI: -0.110,-0.006), as well as screen time (β = -0.100, SE = 0.047, p = 0.036, 95% CI: -0.193, -0.006). Thus, we see that a buildup of too much or too little sleep and sustained higher smartphone use is reflected in differences between people’s perception of their cognitive functioning. For step count, the between-person association remained (β = 0.187, SE = 0.041, p <0.001, 95% CI: 0.108, 0.267), but the within-person association disappeared. This suggests that a weekly average smooths out the effects that were found with the two days window. Therefore, the perceived cognitive benefit of taking more steps than one’s average may be more of a momentary experience.

Together, the differences we find in this sensitivity analysis highlight that behavior–cognition associations are timescale-dependent, though replication with larger samples would help confirm their robustness.

**Supplementary Table 3**. Standardized associations between digital markers of daily life behaviors (sleep duration, step count, and screen time) as predictors of performance-based cognitive smartphone assessments (global performance score of THINC-it^®^ tasks), using behavioral data from one week preceding the cognitive assessments as a sensitivity analysis (instead of two days). Level-1 is the within-person level. Level-2 is the between-person level. Significant associations are in bold. Abbreviations: N = unique participants, t = observations.

| **Model (Predictor – Outcome)**  **Sensitivity analysis: one-week window** | **Sleep duration – Global Performance score**  **(N = 379, t = 1722)** | | | | **Step count – Global Performance score**  **(N = 401, t = 1941)** | | | | **Screen time – Global Performance score**  **(N = 341, t = 1412)** | | | |
| --- | --- | --- | --- | --- | --- | --- | --- | --- | --- | --- | --- | --- |
| **Fixed effects coefficients** | **Estimate (SE)** | **95% CI** | | **p value** | **Estimate (SE)** | **95% CI** | | **p value** | **Estimate (SE)** | **95% CI** | | **p value** |
| Mean/Intercept | **1.378 (0.190)** | **[1.005, 1.753]** | | **<0.001***** | **1.273 (0.175)** | **[0.929, 1.618]** | | **<0.001***** | **1.069 (0.203)** | **[0.671, 1.469]** | | **<0.001***** |
| Time (relative to each person’s start) | **4.97E-04 (7.18E-05)** | **[3.55E-04, 6.38E-04]** | | **<0.001***** | **4.85E-04 (6.71E-05)** | **[3.53E-04, 6.17E-04]** | | **<0.001***** | **6.22E-04 (8.29E-05)** | **[4.59E-04, 7.85E-04]** | | **<0.001***** |
| Behavior within-person component | 0.015 (0.011) | [-0.006, 0.036] | | 0.170 | -0.018 (0.011) | [-0.038, 0.003] | | 0.093^ | 0.006 (0.012) | [-0.018, 0.031] | | 0.608 |
| Behavior within-person component (quadratic) | -0.001 (0.006) | [-0.013, 0.012] | | 0.929 | **-** | **-** | | **-** | **-** | **-** | | **-** |
| Behavior between-person component | -0.002 (0.036) | [-0.074, 0.069] | | 0.949 | **0.099 (0.034)** | **[0.032, 0.167]** | | **0.004**** | 0.038 (0.040) | [-0.041, 0.117] | | 0.344 |
| Behavior between-person component (quadratic) | **-0.073 (0.022)** | **[-0.116, -0.030]** | | **<0.001***** | **-** | **-** | | **-** | **-** | **-** | | **-** |
| Age | **-0.040 (0.002)** | **[-0.045, -0.035]** | | **<0.001***** | **-0.040 (0.002)** | **[-0.044, -0.035]** | | **<0.001***** | **-0.038 (0.003)** | **[-0.043, -0.033]** | | **<0.001***** |
| Gender (ref=male) | -0.031 (0.090) | [-0.209, 0.146] | | 0.728 | -0.020 (0.083) | [-0.184, 0.144] | | 0.813 | -0.011 (0.093) | [-0.195, 0.172] | | 0.904 |
| Years of education | **0.028 (0.007)** | **[0.014, 0.042]** | | **<0.001***** | **0.029 (0.006)** | **[0.016, 0.042]** | | **<0.001***** | **0.039 (0.007)** | **[0.024, 0.054]** | | **<0.001***** |
| **Random effects coefficients** | **Estimate (SD)** | | | | **Estimate (SD)** | | | | **Estimate (SD)** | | | |
| Level-2 error term | 0.424 (0.651) | | | | 0.415 (0.644) | | | | 0.425 (0.652) | | | |
| Level-1 error term | 0.211 (0.459) | | | | 0.211 (0.459) | | | | 0.215 (0.464) | | | |
| **Explained Variance (R^2^)** | **Level** | | **Total** | | **Level** | | **Total** | | **Level** | | **Total** | |
| R^2^ Level-1 | 1.97% | | 0.39% | | 2.31% | | 0.46% | | 3.96% | | 0.82% | |
| R^2^ Level-2 | 52.08% | | 41.87% | | 51.96% | | 41.55% | | 50.02% | | 39.60% | |
| Total R^2^ | 42.26% | | | | 42.02% | | | | 40.42% | | | |

^p<0.1, *p<0.05, **p<0.0083 (corrected alpha for multiple comparisons – α/6), ***p<0.001

**Supplementary Table 4**. Standardized associations between digital markers of daily life behaviors (sleep duration, step count, and screen time) as predictors of self-reported cognitive functioning (PDQ-5 sum score), using behavioral data from one week preceding the cognitive assessments as a sensitivity analysis (instead of two days). Level-1 is the within-person level. Level-2 is the between-person level. Significant associations are in bold. Abbreviations: N = unique participants, t = observations, PDQ-5 = 5-item Perceived Deficits Questionnaire.

| **Model (Predictor – Outcome)**  **Sensitivity analysis: one-week window** | **Sleep duration – PDQ-5 sum score**  **(N = 379, t = 1770)** | | | | **Step count – PDQ-5 sum score**  **(N = 401, 1940)** | | | | **Screen time (predictor) – PDQ-5 sum score**  **(N = 340, t = 1407)** | | | |
| --- | --- | --- | --- | --- | --- | --- | --- | --- | --- | --- | --- | --- |
| **Fixed effects coefficients** | **Estimate (SE)** | **95% CI** | | **p value** | **Estimate (SE)** | **95% CI** | | **p value** | **Estimate (SE)** | **95% CI** | | **p value** |
| Mean/Intercept | **-0.946 (0.233)** | **[-1.404, -0.488]** | | **<0.001***** | **-0.831 (0.208)** | **[-1.239, -0.422]** | | **<0.001***** | **-0.903 (0.240)** | **[-1.373, -0.431]** | | **<0.001***** |
| Time (relative to each person’s start) | -5.68E-05 (7.00E-05) | [-1.94E-04, 8.05E-05] | | 0.417 | -3.11E-05 (6.47E-05) | [-1.58E-04, 9.58E-05] | | 0.631 | -9.77E-05 (7.58E-05) | [-2.46E-04, 5.09E-05] | | 0.198 |
| Behavior within-person component | -0.001 (0.011) | [-0.022, 0.020] | | 0.919 | 0.017 (0.010) | [-0.003, 0.037] | | 0.095^ | **-0.034 (0.011)** | **[-0.055, -0.012]** | | **0.003**** |
| Behavior within-person component (quadratic) | -0.003 (0.006) | [-0.015, 0.009] | | 0.596 | **-** | **-** | | **-** | **-** | **-** | | **-** |
| Behavior between-person component | 0.026 (0.044) | [-0.061, 0.112] | | 0.564 | **0.187 (0.041)** | **[0.108, 0.267]** | | **<0.001***** | **-0.100 (0.047)** | **[-0.193, -0.006]** | | **0.036*** |
| Behavior between-person component (quadratic) | **-0.058 (0.027)** | **[-0.110, -0.006]** | | **0.030*** | **-** | **-** | | **-** | **-** | **-** | | **-** |
| Age | **0.013 (0.003)** | **[0.008, 0.019]** | | **<0.001***** | **0.011 (0.003)** | **[0.006, 0.016]** | | **<0.001***** | **0.009 (0.003)** | **[0.003, 0.016]** | | **0.004**** |
| Gender (ref=male) | -0.091 (0.111) | [-0.309, 0.127] | | 0.412 | -0.148 (0.099) | [-0.343, 0.046] | | 0.135 | -0.039 (0.110) | [-0.256, 0.178] | | 0.725 |
| Years of education | **0.025 (0.009)** | **[0.008, 0.042]** | | **0.003**** | **0.025 (0.008)** | **[0.010, 0.040]** | | **<0.001***** | **0.031 (0.009)** | **[0.013, 0.048]** | | **<0.001***** |
| **Random effects coefficients** | **Estimate (SD)** | | | | **Estimate (SD)** | | | | **Estimate (SD)** | | | |
| Level-2 error term | 0.683 (0.826) | | | | 0.621 (0.788) | | | | 0.640 (0.800) | | | |
| Level-1 error term | 0.197 (0.443) | | | | 0.194 (0.441) | | | | 0.174 (0.417) | | | |
| **Explained Variance (R^2^)** | **Level** | | **Total** | | **Level** | | **Total** | | **Level** | | **Total** | |
| R^2^ Level-1 | 0.10% | | 0.02% | | 0.23% | | 0.05% | | 0.98% | | 0.19% | |
| R^2^ Level-2 | 8.81% | | 6.98% | | 12.82% | | 10.07% | | 9.02% | | 7.22% | |
| Total R^2^ | 7.00% | | | | 10.12% | | | | 7.41% | | | |

^p<0.1, *p<0.05, **p<0.0083 (corrected alpha for multiple comparisons – α/6), ***p<0.001

1. **Reporting the unstandardized coefficients for a more tangible interpretation**

In our main analysis we reported standardized results to compare results across the different behavior-cognition models. Nonetheless, to provide a more tangible interpretation, we include in this section a description of our findings using the unstandardized coefficients. This way, the relationships between the variables can be seen in their original units.

These coefficients can be found in the project’s OSF URL under ‘Results → Main Analysis’ (<https://osf.io/r36mk/>). There are two Excel files there, one for each cognitive outcome, where you will see how we progressively built the models up until finding the best fitting one using unstandardized data points for all variables. The two files are:

- File 1: Summary Analyses Behaviors – Global Performance

Because of its negative quadratic between-person association with sleep duration (Model 6 under the ‘Sleep – Performance’ tab), we found people with one hour above or below the average (i.e., 7.5 hours), performance scores drop 1.11 points for each hour squared (e.g., people with two hours below the average sleep time have 4.48 points lower average performance score (2^2^ x 1.11 = 4.48)). Regarding step count (Model 4 under the ‘Steps – Performance ‘ tab), the between-person association we found shows that for every step more, there is an increase in 0.000491 points in performance. A better way to understand this would be to multiply this 1000, so for every 1000 average steps more, the performance scores are 0.49 higher. If it is 10000 steps (i.e., the default number of recommended steps per day), then scores are 4.9 points higher. Finally, for the between-person association with screen time (Model 4 under the ‘Screens – Performance’ tab), for every minute with the screen unlocked, performance scores are 0.01 higher (i.e., every hour/60 minutes of average screen time is associated with a 0.6 point performance score increase).

- File 2: Summary Analyses Behaviors – PDQ-5

Positive associations were found between self-reported cognitive functioning and step count at both levels of analysis (Model 4 under the ‘Steps – PDQ-5’ tab). We found that for every 1000 average steps more, there is a 0.274 increase in PDQ-5 scores (i.e., 10000 steps = 2.74 points). At the within-person level, on days where someone walked 1000 steps above their average, their PDQ-5 scores were 0.057 points higher. For screen time (Model 4 under the ‘Screens – PDQ-5’ tab), the negative association found at the within-person level indicates that on days where the screen was unlocked one hour above the average, PDQ-5 scores were 0.12 points lower.

1. **References**

Difrancesco, S., Lamers, F., Riese, H., Merikangas, K. R., Beekman, A. T. F., van Hemert, A. M., Schoevers, R. A., & Penninx, B. W. J. H. (2019). Sleep, circadian rhythm, and physical activity patterns in depressive and anxiety disorders: A 2-week ambulatory assessment study. *Depression and Anxiety*, *36*(10), 975–986. <https://doi.org/10.1002/da.22949>

Kalkbrenner, M. T. (2023). Alpha, Omega, and H Internal Consistency Reliability Estimates: Reviewing These Options and When to Use Them. *Counseling Outcome Research and Evaluation*, *14*(1), 77–88. <https://doi.org/10.1080/21501378.2021.1940118>

Kroenke, K., Strine, T. W., Spitzer, R. L., Williams, J. B. W., Berry, J. T., & Mokdad, A. H. (2009). The PHQ-8 as a measure of current depression in the general population. *Journal of Affective Disorders*, *114*(1-3), 163–173. <https://doi.org/10.1016/j.jad.2008.06.026>

Kwak, S. K., & Kim, J. H. (2017). Statistical data preparation: management of missing values and outliers. *Korean Journal of Anesthesiology*, *70*(4), 407–411. <https://doi.org/10.4097/kjae.2017.70.4.407>

Matcham, F., Leightley, D., Siddi, S., Lamers, F., White, K. M., Annas, P., de Girolamo, G., Difrancesco, S., Haro, J. M., Horsfall, M., Ivan, A., Lavelle, G., Li, Q., Lombardini, F., Mohr, D. C., Narayan, V. A., Oetzmann, C., Penninx, B., Bruce, S.,…consortium, R.-C. (2022). Remote Assessment of Disease and Relapse in Major Depressive Disorder (RADAR-MDD): recruitment, retention, and data availability in a longitudinal remote measurement study. *BMC Psychiatry*, *22*(1), 136. <https://doi.org/10.1186/s12888-022-03753-1>

McIntyre, R. S., Best, M. W., Bowie, C. R., Carmona, N. E., Cha, D. S., Lee, Y., Subramaniapillai, M., Mansur, R. B., Barry, H., Baune, B. T., Culpepper, L., Fossati, P., Greer, T. L., Harmer, C., Klag, E., Lam, R. W., Wittchen, H. U., & Harrison, J. (2017). The THINC-Integrated Tool (THINC-it) Screening Assessment for Cognitive Dysfunction: Validation in Patients With Major Depressive Disorder. *J Clin Psychiatry*, *78*(7), 873–881. <https://doi.org/10.4088/JCP.16m11329>

National Multiple Sclerosis Society. (2025). *Perceived Deficits Questionnaire (PDQ)*. Retrieved August from <https://www.nationalmssociety.org/for-professionals/for-researchers/researcher-resources/research-tools/clinical-study-measures/pdq>

Ross-Adelman, M., Aalbers, G., Matcham, F., Simblett, S., Leightley, D., Siddi, S., Haro, J. M., Oetzmann, C., Narayan, V. A., Hotopf, M., Myin-Germeys, I., de Jonge, P., Lamers, F., & Penninx, B. W. J. H. (2025). The Association Between Cognitive Functioning and Depression Severity: A Multiwave Longitudinal Remote Assessment Study. *Depression and Anxiety*, *2025*(1). [https://doi.org/10.1155/da/1509978](https://awspntest.apa.org/doi/10.1155/da/1509978)

Sun, S. X., Folarin, A. A., Zhang, Y. Z., Cummins, N., Garcia-Dias, R., Stewart, C., Ranjan, Y., Rashid, Z., Conde, P., Laiou, P., Sankesara, H., Matcham, F., Leightley, D., White, K. M., Oetzmann, C., Ivan, A., Lamers, F., Siddi, S., Simblett, S.,…Dobson, R. J. B. (2023). Challenges in Using mHealth Data From Smartphones and Wearable Devices to Predict Depression Symptom Severity: Retrospective Analysis. *Journal of Medical Internet Research*, *25*. doi: [10.2196/45233](https://doi.org/10.2196/45233)

Vos, A. L., de Bruijn, G. J., Klein, M. C. A., Boerman, S. C., Stuber, J. M., & Smit, E. G. (2025). Effectiveness of a Just-In-Time Adaptive App to Increase Daily Steps: An RCT. *Am J Prev Med*, *68*(1), 154–163. <https://doi.org/10.1016/j.amepre.2024.09.010>
